# Pairwise Growth Dynamics of Environmental *Escherichia coli* and *Enterococcus* Isolates with Contrasting Antibiotic Susceptibility Phenotypes: Influence of Temperature

**DOI:** 10.3390/antibiotics15090897

**Published:** 2026-09-11

**Authors:** Athanasios Tsiartsafis, Foteini F. Parlapani

**Affiliations:** Department of Ichthyology and Aquatic Environment, School of Agricultural Sciences, University of Thessaly, Fitoko Street, 38446 Volos, Greece; atsiartsafis@uth.gr

**Keywords:** antimicrobial resistance, antibiotic susceptibility phenotype, pairwise growth dynamics, growth kinetics, temperature, predictive microbiology, mussel farming, *Escherichia coli*, *Enterococcus faecalis*, *Enterococcus faecium*

## Abstract

**Background/Objectives:** Environmental bacterial isolates with contrasting, previously established antimicrobial-susceptibility phenotypes may differ in growth under antibiotic-free conditions, but such differences can reflect strain background and temperature. This study characterized the monoculture population development and growth kinetics of predefined pairs of naturally occurring, genetically distinct environmental *Escherichia coli*, *Enterococcus faecalis*, and *Enterococcus faecium* isolates selected from a broader previously characterized collection. **Methods:** Nine isolates were grown separately in a controlled nutrient-enriched seawater model at 12, 22, and 30 °C, representing low, intermediate, and high water-temperature conditions relevant to the Thermaic Gulf. Growth was described using the Baranyi–Roberts model. Within each predefined strain pair and temperature, population development over time was assessed by two-way mixed-design ANOVA with Greenhouse–Geisser correction and Šídák-adjusted time-point comparisons. Temperature dependence of *μ*_max_ was summarized using Ratkowsky and Arrhenius models. **Results:** Population-development patterns differed between paired isolates in all pair–temperature comparisons except MA60–T640 at 30 °C, but the direction and degree of the differences varied among pairs and sampling times. Fitted *μ*_max_ did not always correspond directly to observed population development, as illustrated by SB10–T280. SB10 had a higher *μ*_max_ at all three temperatures while remaining at lower absolute population levels during early-to-mid growth. **Conclusions:** The selected environmental isolate pairs did not show a uniform direction of difference associated with antimicrobial-susceptibility phenotype. Population-development patterns were pair- and time-specific and differed among the tested temperature conditions, while fitted *μ*_max_ did not always reflect observed population development. These findings provide a temperature-resolved basis for interpreting the growth behaviour of selected environmental isolates with contrasting antimicrobial-susceptibility phenotypes under controlled nutrient-enriched marine conditions across temperatures relevant to warming shellfish-production environments.

## 1. Introduction

Antimicrobial resistance (AMR) is a major global health challenge that extends across human, animal, food, and environmental systems. In 2019, bacterial AMR was estimated to be directly responsible for 1.27 million deaths and associated with 4.95 million deaths worldwide [1]. Aquatic environments are integral to this One Health continuum because they receive antibiotic-resistant bacteria and resistance determinants from municipal wastewater, hospitals, animal production, agriculture, and surface runoff and can subsequently transport them toward coastal food-production areas [2,3].

Marine bivalves are particularly relevant at the environment–food interface. Through filter feeding, mussels and other bivalves continuously process surrounding seawater and can concentrate microorganisms and other contaminants in their tissues. A systematic review of 103 studies documented antibiotic-resistant bacteria in marine bivalves from production areas, the environment, and retail, including resistance to critically important antimicrobials among *E. coli* isolates [4]. Bivalves have consequently been proposed as sentinels of multidrug-resistant Enterobacteriaceae in coastal waters [5]. Their broader value as sentinels of changing coastal conditions is also supported by seasonal monitoring studies showing temporal and tissue-specific variation in accumulated environmental contaminants [6]. *Escherichia coli* and enterococci are particularly relevant in this context because they are widely associated with faecal contamination of aquatic and shellfish-associated environments, and antimicrobial-resistant isolates of both bacterial groups have been reported in such settings. Beyond their role as environmental sentinels, bivalves are immunologically active hosts in which interactions with microorganisms are influenced by host defence mechanisms. Mediterranean mussels possess a complex innate immune system involving hemocyte-mediated responses, pattern-recognition mechanisms, mucosal barriers, and antimicrobial effectors [7]. In vertebrates, including terrestrial animals, these evolutionarily conserved innate mechanisms are complemented by antigen-specific adaptive immune responses mediated by specialized lymphocyte receptor systems [8]. This distinction is relevant when considering the extent to which bacterial behaviour observed under controlled in vitro conditions can be extrapolated to persistence within living hosts.

Resistance mechanisms can impose biological costs that, under controlled conditions, may influence bacterial growth in antibiotic-free environments [9,10]. However, resistance-associated changes in growth are not universal and may depend on the resistance mechanism, genetic background, mobile genetic element, nutrient availability, osmotic conditions, and other environmental variables [9,10,11]. Consequently, antimicrobial-susceptibility phenotype alone is insufficient to predict the growth behaviour of an environmental isolate. Importantly, the isolates investigated in the present study are naturally occurring, genetically distinct environmental strains rather than isogenic or near-isogenic counterparts differing specifically in resistance phenotypes. Differences observed within a predefined pair therefore cannot be attributed causally to antimicrobial resistance. In addition, because the isolates were cultured separately, the present experiment characterizes monoculture population development and growth kinetics rather than direct competition between paired isolates.

Temperature adds a further layer of complexity because it is a major determinant of bacterial growth kinetics and may influence the expression of strain-specific physiological differences under antibiotic-free conditions. Experimental evolution studies have shown that acquisition of resistance can alter both growth rate and thermal response [12]. The three experimental temperatures used here were selected to represent distinct thermal conditions relevant to mussel-growing areas of northern Greece: 12 °C represents a low-temperature condition, 22 °C an intermediate condition, and 30 °C a high-temperature condition relevant to severe summer warming. Greek bivalve-production areas have experienced summer seawater temperatures of approximately 30–32 °C, accompanied by prolonged heat stress and major mussel mortalities [13,14]. These temperatures were therefore used as representative controlled experimental conditions rather than as a reconstruction of the complete natural thermal regime.

Despite increasing surveillance of AMR in bivalves and shellfish-growing waters, limited information is available on how environmental isolates with contrasting antimicrobial-susceptibility phenotypes differ in their population development when examined under the same conditions in the absence of antibiotics. The isolates examined here were selected from a broader previously characterized environmental collection on the basis of their contrasting antimicrobial-susceptibility phenotypes. The primary question of the present study was whether, within each predefined pair and at a given temperature, the patterns of population development over time differed between the selected isolates. A second objective was to describe how these pairwise patterns varied across the three tested temperature conditions. This distinction is important because a fitted maximum growth rate describes only one component of bacterial growth and may not fully reflect differences in initial population, lag phase, population development over time, or final population level.

Accordingly, the aim of the present study was to characterize the monoculture population development and growth kinetics of predefined pairs of environmental *Escherichia coli*, *Enterococcus faecalis*, and *Enterococcus faecium* isolates with contrasting, previously established antimicrobial-susceptibility phenotypes at 12, 22, and 30 °C. We hypothesized that the paired isolates could show pair-specific differences in population development and fitted growth parameters under antibiotic-free conditions and that the degree or direction of these differences could vary across the tested temperature conditions, without assuming a uniform direction associated with antimicrobial-susceptibility phenotype. Particular emphasis was placed on distinguishing observed population development over time from fitted maximum growth rate. The study therefore provides a controlled, pair-specific characterization of growth behaviour across thermal conditions relevant to the Thermaic Gulf rather than a causal test of resistance-associated effects on growth.

## 2. Results

### 2.1. Overall Growth Dynamics and Baranyi–Roberts Model Fit

**Figure 1:**
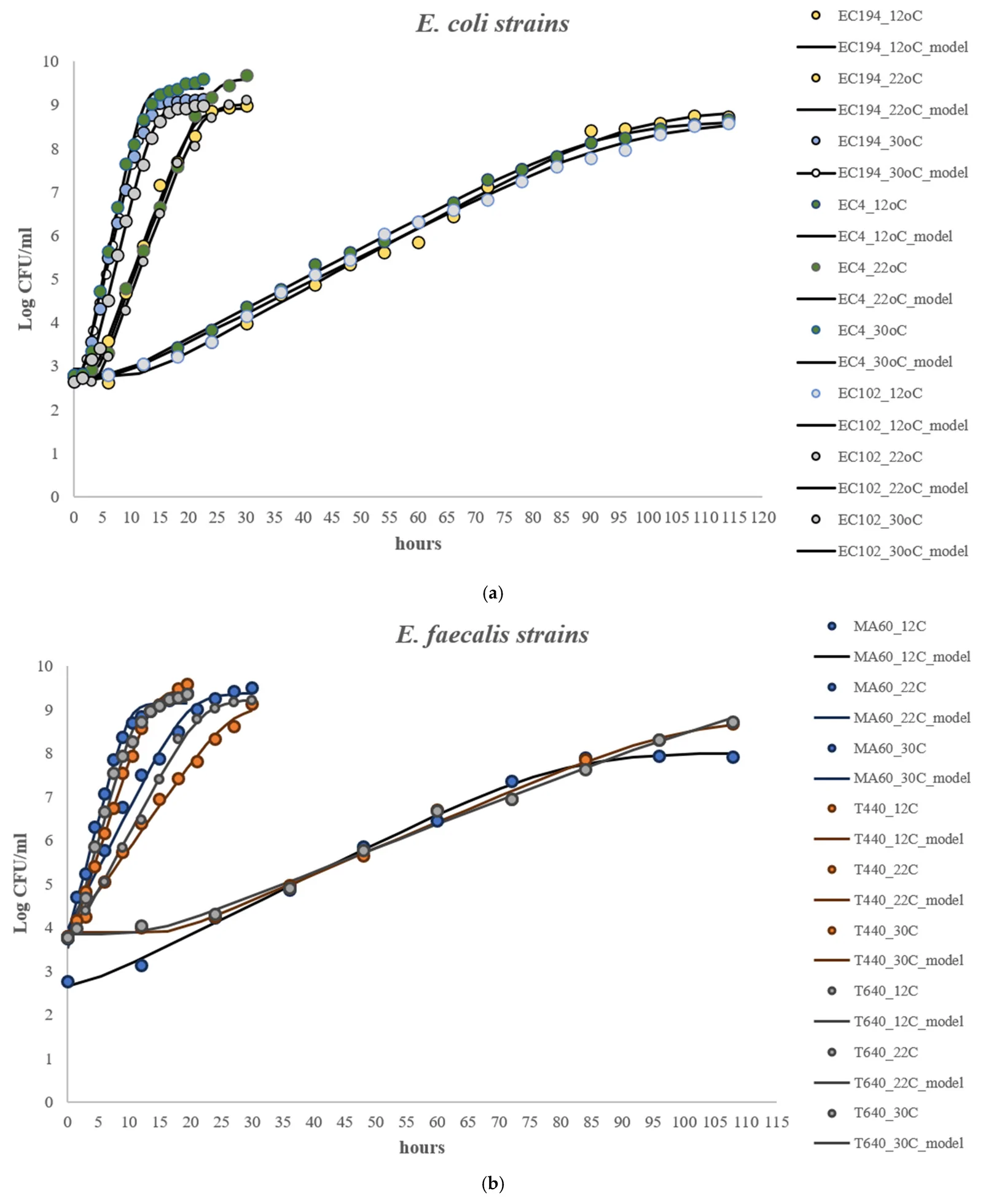

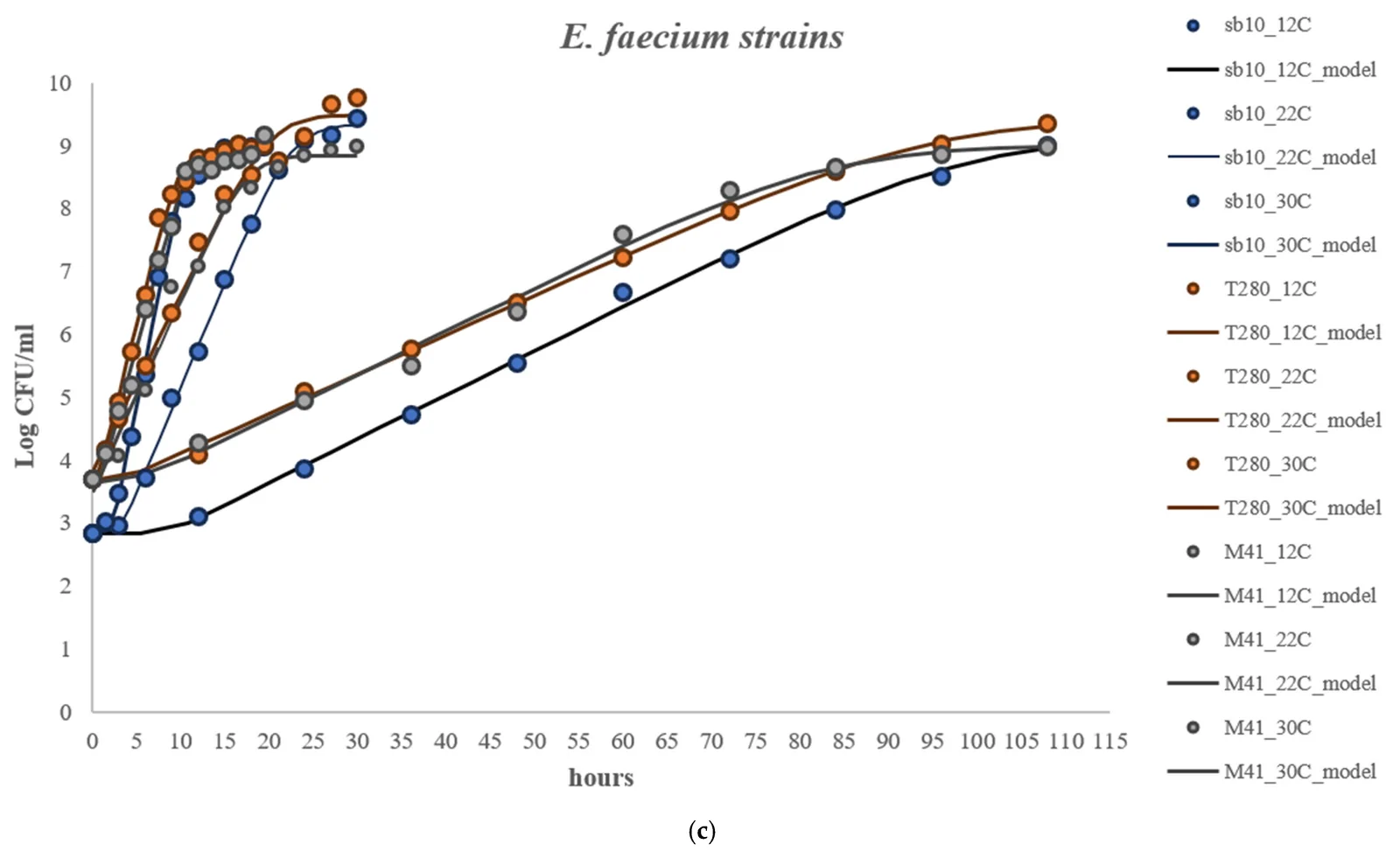
Growth of (**a**) *Escherichia coli* strains EC194, EC4, and EC102, (**b**) *Enterococcus faecalis* strains MA60, T440, and T640, and (**c**) *Enterococcus faecium* strains SB10, T280, and M41 in the nutrient-enriched seawater model at 12 °C, 22 °C, and 30 °C. Symbols represent mean observed populations from three independent experiments, and continuous lines represent the corresponding Baranyi–Roberts model fits.

All nine strains showed clear population increases at 12, 22, and 30 °C (Figure 1). Population development was progressively faster at 22 and 30 °C than at 12 °C, whereas the population levels reached toward the end of incubation varied comparatively less across temperatures. Based on the independent Baranyi–Roberts fits, mean *μ*_max_ values ranged from 0.055–0.072 log_10_ CFU/mL/h at 12 °C, 0.198–0.355 log_10_ CFU/mL/h at 22 °C, and 0.426–0.713 log_10_ CFU/mL/h at 30 °C (Table 1).

Mean fitted lag values ranged from 4.264–17.584 h at 12 °C, 0–4.185 h at 22 °C, and 0–2.480 h at 30 °C. A fitted lag of 0 h represents a valid model estimate and is reported as such. Where lag estimates were available from only one or two of the three independent curve fits, the corresponding effective sample size is indicated explicitly in Table 1 and these estimates are interpreted descriptively.

Estimated initial and final fitted population levels ranged from 2.617 to 3.969 and from 8.015 to 9.613 log_10_ CFU/mL, respectively. Residual-based model assessment indicated generally close agreement between fitted and observed curves, with pooled RMSE values ranging from 0.083 to 0.230 log_10_ CFU/mL. The largest pooled RMSE values were obtained for M41 and T280 at 22 °C (0.230 and 0.229 log_10_ CFU/mL, respectively). Mean R^2^ values ranged from 0.9817 to 0.9985 and are retained as complementary descriptive measures of fit rather than being interpreted independently as demonstrating model adequacy.

### 2.2. Pairwise Growth Dynamics of Escherichia coli Strains

The EC194–EC102 pair was predefined as an ampicillin-resistant versus ampicillin-susceptible comparison, with EC194 and EC102 representing the respective original phenotype designations. The pattern of population development over time differed significantly between the two strains at all three temperatures (Greenhouse–Geisser-corrected *p* = 0.002 at 12 °C, *p* = 0.002 at 22 °C, and *p* = 0.007 at 30 °C; Table S1). At 12 °C, Šídák-adjusted comparisons (Table S2) showed that EC194 populations were significantly lower than those of EC102 at 54 and 60 h but significantly higher at 78, 90, and 96 h, with the largest significant difference observed at 90 h (+0.650 log10 CFU/mL). At 22 °C, EC194 populations were significantly higher at 9 and 15 h, whereas no significant differences were detected at later sampling times; at the final sampling time (30 h), EC102 was numerically higher than EC194 (9.11 versus 8.98 log10 CFU/mL). At 30 °C, EC194 populations were significantly higher from 4.5 to 15 h and again at 19.5 h, with the largest difference observed at 6 h (+0.986 log10 CFU/mL). Baranyi–Roberts modelling yielded a higher *μ*_max_ for EC194 than for EC102 at 30 °C (0.576 versus 0.538 log_10_ CFU/mL/h), together with a shorter estimated lag phase (1.534 versus 2.480 h). At 12 and 22 °C, the corresponding *μ*_max_ values were similar (0.072 versus 0.066 and 0.353 versus 0.352 log_10_ CFU/mL/h, respectively).

The EC4–EC102 pair was predefined as a tetracycline-resistant versus tetracycline-susceptible comparison, with EC4 and EC102 representing the respective original phenotype designations. The pattern of population development over time differed significantly between the two strains at 12 °C (*p* = 0.029), 22 °C (*p* = 0.001), and 30 °C (*p* = 0.002). At 12 °C, population levels were generally similar, with EC4 showing significantly higher populations only at 72 and 96 h. At 22 °C, EC4 populations were significantly higher at 9, 21, 24, and 30 h, with a difference of +0.569 log10 CFU/mL at the final sampling time. The most pronounced separation was observed at 30 °C, where EC4 populations were significantly higher than those of EC102 at every sampling time from 4.5 to 22.5 h, with the largest difference observed at 4.5 h (+1.329 log10 CFU/mL). The two strains showed similar *μ*_max_ values at 12 and 22 °C (0.069 versus 0.066 and 0.355 versus 0.352 log10 CFU/mL/h, respectively), whereas at 30 °C *μ*_max_ was higher for EC4 than for EC102 (0.629 versus 0.538 log10 CFU/mL/h). EC4 also showed a shorter estimated lag phase and a higher fitted final population level at 30 °C.

### 2.3. Pairwise Growth Dynamics of Enterococcus faecalis Strains

The MA60–T440 and MA60–T640 pairs were predefined as vancomycin-resistant versus vancomycin-susceptible comparisons, with MA60 representing the resistant-designated isolate and T440 and T640 the susceptible-designated comparators in the original study design. At 12 °C, the initial population of MA60 was approximately 2.75 log10 CFU/mL, around 1 log10 CFU/mL lower than those of T440 and T640 (approximately 3.78 and 3.77 log10 CFU/mL, respectively). The pattern of population development over time differed significantly between MA60 and T440 and between MA60 and T640 (both *p* < 0.001; Table S1). MA60 remained significantly lower at 12 h, whereas the direction of the pairwise differences subsequently changed. At 72 h, MA60 populations were significantly higher than those of both comparator strains, whereas at 108 h they were again lower (Table S2). Thus, the relative population levels within both pairs varied over time against the background of the initial difference in population abundance. At 22 °C, the three *E. faecalis* strains had comparable initial population levels. The pattern of population development over time differed significantly between MA60 and T440 (*p* = 0.001) and between MA60 and T640 (*p* = 0.002). MA60 populations were significantly higher than those of T440 at several sampling times between 3 and 21 h and again at 27 h, with the largest observed difference at 21 h (+1.209 log10 CFU/mL). Relative to T640, MA60 showed significantly higher populations at 6, 9, 12, and 21 h. The fitted *μ*_max_ of MA60 (0.275 log_10_ CFU/mL/h) was higher than that of T440 (0.198 log_10_ CFU/mL/h) and slightly higher than that of T640 (0.261 log_10_ CFU/mL/h).

At 30 °C, the pattern of population development over time also differed significantly between MA60 and T440 (*p* = 0.003). MA60 populations were significantly higher at 3, 6, 7.5, 9, and 10.5 h, whereas T440 reached a higher final observed population. For MA60–T640, no significant difference in the pattern of population development over time was detected after Greenhouse–Geisser correction (*p* = 0.055). Across the three tested temperatures, MA60 showed a higher fitted *μ*_max_ than both comparator strains, whereas the observed pairwise differences in population levels varied across temperatures and sampling times.

### 2.4. Pairwise Growth Dynamics of Enterococcus faecium Strains

The SB10–T280 pair was predefined as a vancomycin-resistant versus vancomycin-susceptible comparison, with SB10 and T280 representing the respective original phenotype designations. Population development over time differed significantly between the two strains at 12 °C (*p* = 0.002), 22 °C (*p* < 0.001), and 30 °C (*p* < 0.001; Table S1). The initial population of T280 was approximately 0.87 log10 CFU/mL higher than that of SB10 at all three temperatures, and this initial difference contributed to the lower absolute population levels observed for SB10 during the early part of growth. At 12 °C, significant differences were observed at 0, 12, 24, 36, 48, 72, and 108 h; at 22 °C, SB10 populations were significantly lower from 0 to 18 h; and at 30 °C, they were significantly lower from 0 to 9 h (Table S2). The largest observed differences were −1.233, −1.786, and −1.449 log_10_ CFU/mL at 12, 22, and 30 °C, respectively. In contrast, Baranyi–Roberts modelling yielded a higher *μ*_max_ for SB10 at all three temperatures (0.070 versus 0.063, 0.335 versus 0.281, and 0.713 versus 0.557 log_10_ CFU/mL/h, respectively). Thus, the fitted *μ*_max_ values did not correspond directly to the observed differences in absolute population levels over time. The M41–T280 pair was predefined as a tetracycline-resistant versus tetracycline-susceptible comparison, with M41 and T280 representing the respective original phenotype designations. Initial population levels were similar, while the pattern of population development over time differed significantly between the two strains at all three temperatures (all *p* < 0.001). At 12 °C, the direction of the significant differences changed over time: M41 populations were lower at 48 h, higher at 72 h, and lower again at 108 h. At 22 °C, T280 populations were significantly higher at 3, 6, 12, 24, 27, and 30 h, whereas M41 populations were significantly higher at 9 h; at the final sampling time, M41 was 0.794 log10 CFU/mL lower than T280. At 30 °C, T280 populations were significantly higher at 4.5, 7.5, and 9 h, whereas no significant differences were detected at subsequent sampling times. At 19.5 h, M41 was numerically higher than T280 (9.17 versus 9.00 log10 CFU/mL), but this difference was not significant after Šídák adjustment. Baranyi–Roberts modelling yielded similar *μ*_max_ values for M41 and T280 at 12 and 22 °C, whereas at 30 °C *μ*_max_ was lower for M41 than for T280 (0.524 versus 0.557 log10 CFU/mL/h). These results further illustrate that differences in fitted maximum growth rate and observed population development over time were not necessarily concordant.

### 2.5. Exploratory Secondary Models of Temperature Dependence

**Figure 2:**
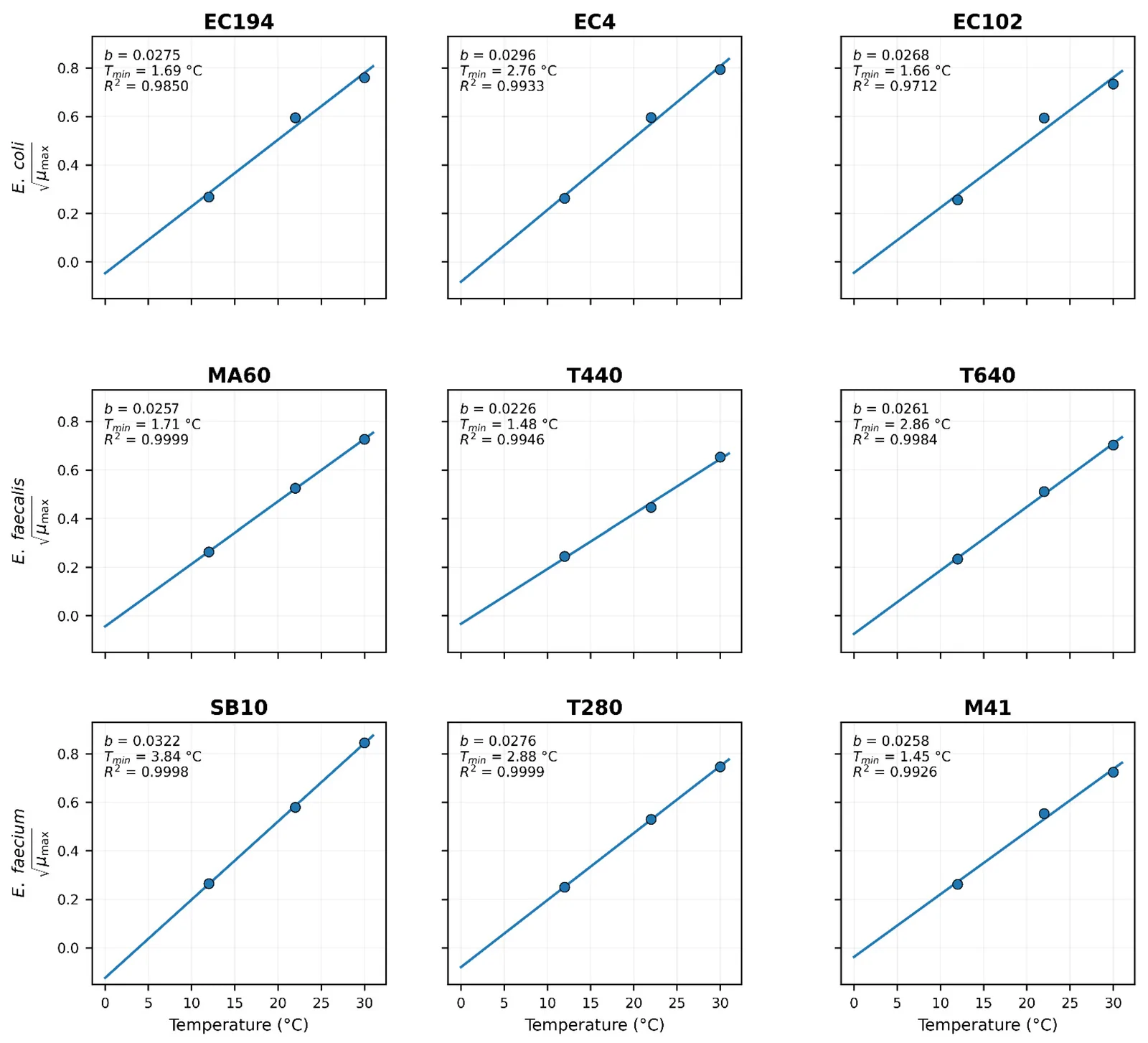
Ratkowsky square-root model fits for the nine strains. Points represent the square root of the mean *μ*_max_ at 12, 22, and 30 °C; lines represent fitted relationships. *T*_min_ is the theoretical x-intercept and should not be interpreted as an experimentally established minimum growth temperature.

The Ratkowsky square-root model was used to summarize the relationship between temperature and √ *μ*_max_, with *R*^2^ values ranging from 0.9712 to 0.9999 and RMSE values from 0.00171 to 0.03399 (Figure 2; Table 2 and Table S3). Estimated *b* values ranged from 0.0226 to 0.0322, while theoretical *T*_min_ values ranged from 1.45 to 3.84 °C. Among the predefined strain pairs, the point estimate of *b* was higher for EC4 than for EC102 (0.0296 versus 0.0268), similar for EC194 and EC102 (0.0275 versus 0.0268), higher for MA60 than for T440 but similar to that of T640 (0.0257 versus 0.0226 and 0.0261, respectively), higher for SB10 than for T280 (0.0322 versus 0.0276), and lower for M41 than for T280 (0.0258 versus 0.0276). These parameters summarize the temperature dependence of fitted *μ*_max_ and do not represent differences in population development over the complete growth period. Because each model was fitted using only three temperature points, the high *R*^2^ values should not be interpreted as demonstrating model robustness or validated predictive performance. Theoretical *T*_min_ values are extrapolated x-intercepts rather than experimentally established minimum growth temperatures, and the broad confidence intervals for several estimates further limit precise biological interpretation.

**Figure 3:**
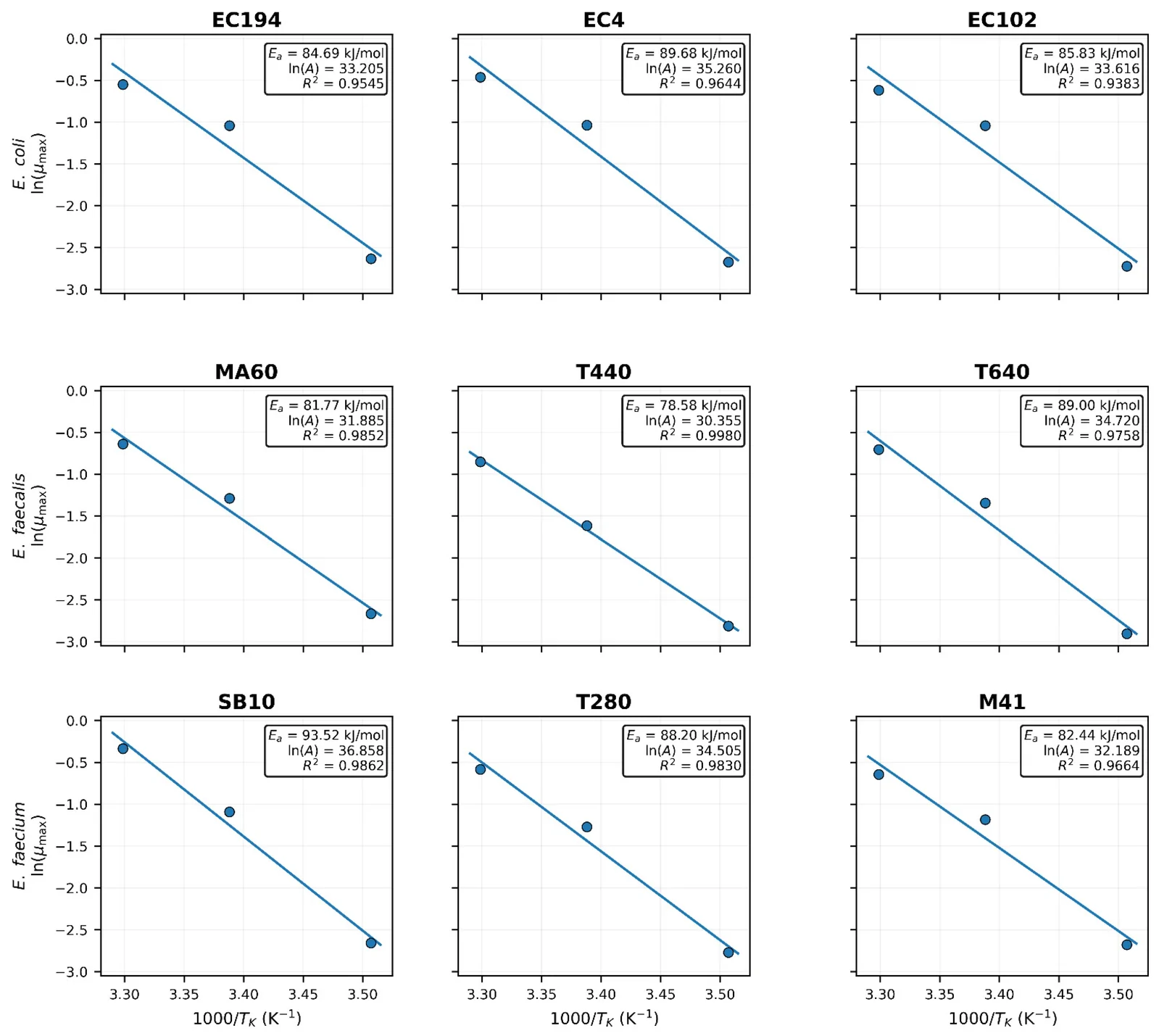
Linearized Arrhenius model fits for the nine strains. Points represent ln(*μ*_max_) at 12, 22, and 30 °C; lines represent fitted relationships. *E*_a_ values are apparent activation energies derived from three temperature points.

The linearized Arrhenius model was used to summarize the temperature dependence of fitted *μ*_max_, yielding *R*^2^ values ranging from 0.9383 to 0.9980 and RMSE values from 0.0364 to 0.2257 on the ln(*μ*_max_) scale (Figure 3; Table 3 and Table S4). Apparent activation-energy (*E*_a_) estimates ranged from 78.58 to 93.52 kJ/mol. Among the predefined strain pairs, the point estimate of apparent *E*_a_ was higher for EC4 than for EC102, higher for MA60 than for T440 but lower than for T640, higher for SB10 than for T280, and lower for M41 than for T280. These relative patterns were generally consistent with those observed for the Ratkowsky *b* estimates. Because each model was based on only three temperature points and several confidence intervals were very broad, the apparent *E*_a_ values are interpreted only as descriptive indices of temperature dependence rather than as precise estimates of an underlying mechanistic temperature-response parameter. Accordingly, the high *R*^2^ values obtained from these three-point relationships are not interpreted as demonstrating model robustness or validated predictive performance.

## 3. Discussion

The principal finding of this study is that the selected environmental strain pairs with contrasting antimicrobial-susceptibility phenotypes did not exhibit a single, predictable pattern of monoculture population development in the absence of antibiotics. The pattern of population development over time differed significantly between the paired strains in all pair–temperature comparisons except MA60–T640 at 30 °C, but the direction and degree of the differences varied among strain pairs and sampling times. Because the compared strains are naturally occurring, genetically distinct environmental isolates, these observations cannot be attributed causally to antimicrobial resistance itself. Moreover, the statistical analyses were performed separately within each temperature; therefore, differences among the three temperature-specific patterns are interpreted descriptively and not as demonstrating a formally tested strain × temperature interaction.

An important finding was the distinction between observed population development, fitted kinetic parameters, and initial population abundance. *μ*_max_ describes the steepest fitted phase of population increase, whereas the population observed at a given sampling time also reflects the starting population, lag behaviour, and progression toward the final population level. The SB10–T280 comparison illustrates this distinction particularly clearly. Although SB10 showed a higher fitted *μ*_max_ at all three temperatures, its absolute population remained lower than that of T280 during substantial portions of early-to-mid growth. This pattern occurred against an initial difference of approximately 0.87 log_10_ CFU/mL, with T280 starting at the higher population level, together with differences in lag behaviour. Similarly, at 12 °C, MA60 started approximately 1 log_10_ CFU/mL below T440 and T640 but subsequently reached higher population levels than both comparator strains at selected sampling times. These findings emphasize that differences in absolute population abundance at individual sampling times should be interpreted in the context of the experimentally observed starting populations and should not be considered interchangeable with differences in fitted *μ*_max_.

The other predefined comparisons reinforced the same principle. At 12 °C, EC194 populations were lower than those of EC102 during part of growth but became higher at later sampling times, demonstrating that even the direction of a pairwise population difference can change during incubation. At 22 °C, differences between EC194 and EC102 occurred at selected sampling times, whereas at 30 °C EC194 showed higher populations during much of active growth together with a modestly higher *μ*_max_. The EC4–EC102 comparison showed comparatively limited population separation at 12 °C, greater separation at selected sampling times at 22 °C, and the most pronounced separation at 30 °C. The *E. faecalis* and *E. faecium* comparisons likewise showed pair-specific combinations of initial abundance, population development, fitted growth rate, and final population level. Taken together, these results indicate that no single kinetic descriptor adequately represents the complete population-development pattern of the selected isolates.

Previous experimental studies have shown that growth effects associated with antimicrobial resistance are not uniform and may depend on the specific resistance determinant or mutation, bacterial genetic background, expression state, mobile genetic element context, and growth environment [15,16,17,18,19,20]. Such findings provide relevant biological context for the heterogeneity observed here, but they should not be interpreted as mechanistic explanations for the present strain-specific patterns. The resistance determinants, mutations, expression states, and mobile genetic elements responsible for the susceptibility phenotypes of the isolates examined here were not characterized as part of this growth-kinetics experiment. The six strain pairs had been predefined on the basis of antimicrobial-susceptibility designations established during the original characterization of the environmental isolate collection. These original designations are retained here to describe the experimental design under which the growth study was conducted. For transparency, the archived disk-diffusion measurements and their interpretation against the current CLSI M100 criteria used in the present revision are reported separately in Supplementary Table S6. Because the paired isolates are genetically distinct environmental strains, the comparisons cannot isolate the contribution of an individual resistance determinant or antimicrobial-susceptibility phenotype. The resistant and susceptible designations used here should therefore be understood strictly as phenotypic descriptors of the predefined experimental pairs rather than as mechanistic genetic categories. Disk-diffusion phenotype alone cannot distinguish resistance-associated effects from unrelated genomic, physiological, or strain-background variation. Consequently, the present growth-kinetics data do not establish that the observed differences in growth were caused by antimicrobial resistance.

Temperature strongly influenced the rate of population development of all nine isolates, with faster growth at 22 and 30 °C than at 12 °C. At the same time, the magnitude and direction of several pairwise differences varied across the three tested temperature conditions. For example, the separation between EC4 and EC102 was most pronounced at 30 °C, while SB10 maintained a higher fitted *μ*_max_ than T280 at all three temperatures despite lower absolute population levels during much of early-to-mid growth. M41 and T280 showed yet another pattern, with relatively small and directionally changing differences at 12 °C and different temporal patterns at 22 and 30 °C. These observations support interpretation of temperature as an important environmental determinant of strain-specific growth behaviour rather than assuming a fixed relationship between antimicrobial-susceptibility phenotype and bacterial population development. Because the statistical analyses were conducted independently within each temperature, however, differences among temperature-specific patterns remain descriptive rather than evidence of a formally tested interaction. Sampling intervals were also adapted to the expected rate of growth at each temperature; consequently, the number or apparent duration of significant sampled time points should not be compared directly across temperatures.

The nutrient-enriched seawater model was designed to provide standardized marine conditions with sufficient nutrient availability for characterization of growth kinetics. It was not intended to reproduce the complete nutritional, physicochemical, microbial, or biological complexity of natural mussel-growing waters. Natural coastal systems include variable nutrient availability, resident microbial communities, competition, predation, fluctuating physicochemical conditions, environmental contaminants, and other interacting factors. In living mussels, bacterial persistence is additionally influenced by filter-feeding dynamics, tissue-associated physicochemical conditions, resident microbiota, innate host immune defences, and other host-associated biological processes. Consequently, the relative growth patterns observed under the present in vitro conditions should not be interpreted as direct predictors of the persistence or relative success of the same isolates within living mussels. At the same time, the filter-feeding behaviour of mussels and their capacity to accumulate environmental inputs support their broader role as biological sentinels of changing coastal conditions [6]. The present experimental system should therefore be interpreted as a controlled model of growth potential under nutrient-enriched marine conditions rather than as a direct simulation of bacterial persistence, community behaviour, or interactions in natural shellfish-production environments.

The Ratkowsky and Arrhenius analyses were retained as exploratory descriptive summaries of the temperature dependence of fitted *μ*_max_ within the tested temperature range. The relative differences in Ratkowsky *b* point estimates among several predefined pairs were broadly reflected in the apparent activation-energy estimates obtained from the Arrhenius analysis. These secondary models must nevertheless be interpreted cautiously because each relationship was fitted using only three temperature points. The broad confidence intervals surrounding several theoretical *T*_min_ and apparent *E*_a_ estimates preclude precise interpretation of thermal limits or mechanistic temperature-response parameters. Likewise, the high *R*^2^ values obtained from three-point relationships should not be interpreted as demonstrating model robustness or validated predictive performance. Their role in the present study is therefore limited to descriptive comparison among the selected isolates, and the numerical *T*_min_ and apparent *E*_a_ estimates should not be overinterpreted. The scope of inference is defined by the controlled experimental design. The three independent experimental runs performed for each strain–temperature condition constituted the experimental units, while repeated sampling times represented longitudinal observations within each run. The study was designed to characterize the population development and fitted growth kinetics of the selected isolates under defined conditions rather than to estimate variability across a broader population of environmental isolates. Likewise, because each strain was cultured separately, the experiment did not assess direct competition between paired isolates. Different experimental questions would require correspondingly different designs. Establishing a causal effect of a specific resistance determinant on growth would require genetically matched or isogenic systems together with molecular characterization of the relevant determinant. Assessing direct interactions between isolates would require co-culture experiments. Validated prediction of temperature dependence would require additional temperature levels, while extrapolation to natural shellfish-production systems would require experiments incorporating natural seawater, resident microbial communities, and/or host-associated conditions. Larger isolate collections would be appropriate where the objective is population-level generalization rather than characterization of predefined strain pairs.

Within these boundaries, the present findings demonstrate that selected environmental isolates with contrasting antimicrobial-susceptibility phenotypes can show markedly different patterns of monoculture population development under the same antibiotic-free conditions, but these differences do not follow a uniform direction associated with susceptibility phenotype. Their interpretation depends on the strain pair, sampling time, initial abundance, fitted kinetic parameter, and tested temperature condition. The findings therefore provide a temperature-resolved basis for interpreting the growth behaviour of selected environmental bacterial isolates under thermal conditions relevant to shellfish-production environments, while maintaining a clear distinction between observed pair-specific growth behaviour and causal resistance-associated effects.

## 4. Materials and Methods

### 4.1. Bacterial Strains, Identification, and Antimicrobial-Susceptibility Phenotypes

Nine environmental isolates were selected from a substantially broader strain collection established during previous microbiological investigations of mussel-farming areas and surrounding potential contamination sources in the Thermaic Gulf, northern Greece. The broader collection included isolates recovered from mussel flesh, mussel-farm water, sediment, wastewater-treatment facilities, and other surrounding environmental sources. Its occurrence, source distribution, species identification, and antimicrobial-susceptibility characterization are reported separately in Tsiartsafis et al. [21].

The present study was designed as a subsequent, focused growth-kinetics experiment. The nine isolates were selected from the previously characterized collection on the basis of their established antimicrobial-susceptibility phenotypes in order to define the predefined strain comparisons examined here (Table 4). The paired isolates are naturally occurring, genetically distinct environmental strains rather than isogenic or near-isogenic counterparts differing only in antimicrobial-susceptibility phenotype.

Species identity had previously been established as part of the characterization of the source collection using high-resolution melting (HRM) analysis of V3–V4 16S rRNA gene amplicons, followed by Sanger sequencing for sequence-based identification. The individual isolation source, sequence identity, and accession number of the closest GenBank sequence match for each of the nine isolates are provided in Supplementary Table S5. These identification procedures were not repeated as part of the present growth-kinetics experiment.

Antimicrobial susceptibility had previously been assessed by disk diffusion during characterization of the broader environmental isolate collection. Briefly, each isolate was inoculated into 10 mL of Tryptic Soy Broth (TSB) and incubated at 37 °C for 24 h. An inoculum from each culture was subsequently spread onto dried Mueller–Hinton agar plates to obtain an approximate initial population of 10^7^ CFU. Twenty commercial antimicrobial disks (Oxoid) were examined. The focal disks relevant to the predefined comparisons in the present growth-kinetics study were ampicillin (10 μg), tetracycline (30 μg), and vancomycin (30 μg). Each antimicrobial disk was applied in duplicate, and plates were incubated at 37 °C for 24 h. Each inhibition zone was measured along three diameters on each of two replicate plates, providing six measurements per isolate–antimicrobial combination. Mean inhibition-zone diameters and standard deviations were calculated from these six measurements.

The archived inhibition-zone measurements were originally recorded in centimetres and are retained in Supplementary Table S6 in their original centimetre units. For numerical comparison with the applicable current CLSI M100 zone-diameter criteria, both mean values and corresponding standard deviations were converted to millimetres by multiplying by 10. The original phenotype designations used to define the growth-kinetics comparisons are retained as study-design descriptors. For transparency in the present revision, the archived focal inhibition-zone measurements are additionally presented alongside the applicable current Clinical and Laboratory Standards Institute (CLSI) M100 disk-diffusion interpretive criteria (36th ed., 2026). These current criteria are reported as a reference framework for interpretation of the archived measurements and are not used retrospectively to redefine which strain pairs were examined experimentally. *Escherichia coli* ATCC 25922 and *Enterococcus faecalis* ATCC 29212 were used as reference control strains for *E. coli* and *Enterococcus* spp., respectively. Supplementary Table S6 provides the archived disk-diffusion measurements for the focal strain–antimicrobial combinations used in the six predefined comparisons, together with disk contents, the original phenotype designation, the applicable current CLSI M100 interpretive criterion, and the corresponding current zone-based classification. MICs and the specific resistance genes, mutations, plasmid structures, or expression states responsible for the observed susceptibility phenotypes were not determined as part of the present growth-kinetics study and are therefore not inferred from the growth data.

### 4.2. Preparation of Inocula

Strains were maintained at −80 °C in a cryoprotective medium until use. Before each experiment, cultures were revived by streaking onto Tryptone Soy Agar (TSA; Neogen, Lansing, MI, USA) and incubating at 37 °C for 24 h. A single well-isolated colony was transferred to 9 mL of Tryptone Soy Broth (TSB; Neogen) and incubated at 37 °C for 24 h. Culture populations were determined by plate counting, and decimal dilutions were prepared in Maximum Recovery Diluent (MRD; Neogen) to obtain a target initial population of approximately 3 log_10_ CFU/mL in the experimental substrate. This value represented a predefined target rather than an imposed identical starting population. An exactly identical viable population was not imposed because the study was designed to characterize the observed population development of each isolate following preparation to the same target inoculum and subsequent exposure to identical environmental conditions, rather than population change relative to an identical baseline. The experimentally observed time-zero populations were therefore retained as measured and were not normalized or otherwise adjusted.

### 4.3. Nutrient-Enriched Seawater Model and Experimental Design

TSB (Neogen, Lansing, MI, USA) was prepared at 30 g/L using seawater instead of deionized water. According to the manufacturer’s formulation, the medium contained, per litre, 17.0 g enzymatic digest of casein, 3.0 g enzymatic digest of soybean, 5.0 g sodium chloride, 2.5 g dipotassium hydrogen phosphate, and 2.5 g glucose monohydrate. The use of a nutrient-rich system was intentional, as mussel-farming areas of the Thermaic Gulf are influenced by riverine discharge and multiple terrestrial and anthropogenic inputs, including wastewater-treatment effluents, agricultural runoff, and drainage or pumping-station discharges, which can locally increase the availability of organic matter and nutrients in coastal waters. The present system was therefore designed to provide a reproducible high-resource marine condition for comparing strain-specific growth behaviour under otherwise standardized conditions. It was not intended to reproduce the exact chemical composition or nutrient concentrations of these environmental inputs, nor the resident microbial communities or broader physicochemical and biological complexity of natural mussel-growing waters. The sterilized substrate was distributed aseptically into 50 mL centrifuge tubes at 40 mL per tube. Each strain was tested separately. Following inoculation, the tubes were carefully mixed to ensure uniform cell distribution and incubated at 12, 22, or 30 °C. Three independent experimental runs were performed for each strain–temperature condition. The selected temperatures represented cool-season conditions (12 °C), warmer seasonal conditions (22 °C), and a high-temperature condition (30 °C) relevant to severe summer warming events documented in Greek mussel-farming areas [13,14]. These temperatures were used as representative controlled experimental conditions rather than as a reconstruction of the complete natural thermal regime.

### 4.4. Sampling and Microbiological Enumeration

Sampling frequency was adapted to the expected growth rate at each temperature. At 12 °C, *E. coli* was sampled every 6 h from 0 to 114 h, whereas *E. faecalis* and *E. faecium* were sampled every 12 h from 0 to 108 h. At 22 °C, all strains were sampled every 3 h from 0 to 30 h. At 30 °C, samples were collected every 1.5 h; monitoring continued to 22.5 h for *E. coli* and to 19.5 h for the enterococci. The different sampling intervals were selected to resolve the markedly different rates of population development at the three temperatures; consequently, the number or apparent duration of significant sampled time points was not considered directly comparable across temperatures.

At each sampling time, samples were serially diluted in Maximum Recovery Diluent (MRD), and 0.1 mL aliquots of the appropriate dilution were surface-plated on Tryptone Soy Agar (TSA) in duplicate. Plates were incubated at 37 °C for 24 h, and plates containing 30–300 colonies were used for population estimation. Counts from the duplicate plates were used to calculate bacterial populations, expressed as log10 colony-forming units per millilitre (log_10_ CFU/mL). Duplicate plates represented technical replicates of the same microbiological determination and were not treated as independent experimental replicates.

### 4.5. Primary Growth Modeling

Each independent growth curve was fitted with the Baranyi–Roberts primary model [22] using DmFit software (Institute of Food Research, Reading, UK). The model yielded the initial population (*y*_0_), maximum growth rate (*μ*_max_), lag time, and final population (*y*_end_). Means and standard deviations were calculated from the three independent fits.

Model fit was evaluated using root mean square error (RMSE) together with the coefficient of determination (R^2^). Pooled RMSE, used as the primary residual-based summary of model error, was calculated from the residual error of all three fitted curves as:

RMSE = √[Σ_i_ SE_i_^2^(n_i_ − p_i_)/Σ_i_ n_i_],

where SE_i_ is the residual standard error of fit i, n_i_ is the number of observations, and p_i_ is the number of fitted parameters.

### 4.6. Secondary Temperature Models

The temperature dependence of *μ*_max_ was first described using the Ratkowsky square-root model [23]:

√*μ*_max_ = *b*(*T* − *T*_min_),

where *b* is the slope parameter, *T* is the incubation temperature (°C), and *T*_min_ is the theoretical temperature corresponding to the x-intercept of the fitted relationship, where √ *μ*_max_ = 0.

Temperature dependence was additionally described using the linearized Arrhenius relationship:

ln(*μ*_max_) = ln(*A*) − *E*_a_/(*RT*_K_),

where *A* is the apparent pre-exponential factor, *E*_a_ is the apparent activation energy (J/mol), *R* is 8.314 J mol^−1^ K^−1^, and *T*_K_ is absolute temperature. Because each secondary model was fitted using only three temperature points, these analyses were considered exploratory and descriptive. Theoretical *T*_min_ and apparent *E*_a_ values were therefore not interpreted as validated biological thresholds or precise mechanistic parameters. Approximate 95% confidence intervals were obtained from the parameter covariance matrix.

### 4.7. Statistical Analysis and Data Treatment

Statistical analyses and secondary growth-model calculations were performed in Python 3.13.5 using NumPy 2.3.5 and SciPy 1.17.0. For each predefined strain pair, observed log_10_ CFU/mL values were analysed separately at each incubation temperature using a two-way mixed-design ANOVA, with strain as the between-subject factor and time as the repeated-measures factor. Each of the three independent experimental replicates was treated as a subject followed across sampling times. Within each pair–temperature analysis, the principal inferential test was the strain × time interaction, which assessed whether the temporal pattern of population development differed between the paired strains. The reported F statistics and Greenhouse–Geisser-corrected *p*-values therefore correspond to the strain × time interaction rather than to the main effect of strain. Greenhouse–Geisser correction was applied to repeated-measures effects to account for departures from sphericity. Šídák-adjusted comparisons were performed between the paired strains at individual sampling times, with the family of comparisons defined as all sampling times within the corresponding pair–temperature analysis. Statistical significance was assessed at α = 0.05. The three independent experimental runs constituted the experimental units for each strain–temperature condition. Repeated sampling times within each run were treated as longitudinal observations of the same experimental unit and not as additional independent replicates. Statistical inference was therefore restricted to the selected isolates and experimental conditions examined.

Ratkowsky square-root models were fitted by nonlinear least squares using scipy.optimize.curve_fit, whereas linearized Arrhenius relationships were estimated using scipy.stats.linregress. Because each secondary model was based on only three temperatures per strain, the resulting parameter estimates were treated as descriptive rather than inferential. Approximate 95% confidence intervals for the Ratkowsky parameters were obtained from the parameter covariance matrix. Complete mixed-design ANOVA outputs and Šídák-adjusted time-point comparisons are provided in Supplementary Tables S1 and S2, respectively. Because temperature was not included as a factor in a single repeated-measures model, differences among temperature-specific pairwise patterns are interpreted descriptively rather than as a formally tested strain × temperature interaction.

### 4.8. Use of Generative Artificial Intelligence in Manuscript Preparation

OpenAI ChatGPT (GPT-5.6 Sol) was used during manuscript preparation and revision to assist with English-language drafting and structural organisation and to support verification of statistical calculations based exclusively on the experimental dataset generated by the authors. The tool was not used to generate experimental data or to make independent methodological, statistical, or scientific decisions. All statistical procedures, analytical choices, numerical outputs, and interpretations were reviewed, verified, and critically evaluated by the authors. The final manuscript was reviewed and approved by all authors, who take full responsibility for its scientific content.

## 5. Conclusions

Across the selected environmental *E. coli*, *E. faecalis*, and *E. faecium* strain pairs, contrasting antimicrobial-susceptibility phenotypes were not associated with a uniform direction of difference in monoculture population development. Instead, pairwise differences varied among strain pairs and sampling times, with the observed patterns also differing across the tested temperature conditions, while fitted *μ*_max_ did not always correspond directly to observed population levels. Differences in initial population levels in selected comparisons further emphasize the need to distinguish starting abundance from subsequent population development and fitted kinetic parameters.

Ratkowsky and Arrhenius modelling provided descriptive representations of the temperature dependence of *μ*_max_, but the three-temperature design and broad confidence intervals limit precise interpretation of thermal-response parameters. Because the isolates were naturally occurring, genetically distinct environmental strains, the observed pairwise differences cannot be attributed causally to antimicrobial resistance. Furthermore, because the isolates were grown separately, the present experiments cannot determine which isolate would predominate during direct co-culture. The study therefore provides a controlled, temperature-resolved characterization of pair-specific growth behaviour under nutrient-enriched seawater conditions rather than a direct prediction of bacterial behaviour in natural shellfish-production environments.

## Figures and Tables

**Table 1:** Primary growth parameters of the nine strains in the nutrient-enriched seawater model, as estimated using the Baranyi–Roberts model.

Strain	*T* (°C)	Lag (h)	*μ*_max_ (log_10_ CFU/mL/h)	*y*_0_ (log_10_ CFU/mL)	*y*_end_ (log_10_ CFU/mL)	*R* ^2^	RMSE
EC194	12	12.188 ± 2.997	0.072 ± 0.003	2.761 ± 0.039	8.910 ± 0.115	0.9939	0.154
EC194	22	3.935 ± 0.245	0.353 ± 0.003	2.937 ± 0.071	8.919 ± 0.007	0.9934	0.158
EC194	30	1.534 ± 0.097	0.576 ± 0.008	2.751 ± 0.013	9.089 ± 0.021	0.9985	0.083
EC4	12	7.782 ± 0.782	0.069 ± 0.001	2.793 ± 0.019	8.644 ± 0.097	0.9979	0.085
EC4	22	4.182 ± 0.035	0.355 ± 0.002	2.846 ± 0.020	9.613 ± 0.119	0.9967	0.123
EC4	30	1.570 ± 0.022	0.629 ± 0.007	2.756 ± 0.043	9.383 ± 0.047	0.9944	0.167
EC102	12	8.360 ± 0.608 *	0.066 ± 0.004	2.617 ± 0.156	8.653 ± 0.272	0.9943	0.140
EC102	22	4.185 ± 0.358	0.352 ± 0.011	2.633 ± 0.127	9.023 ± 0.100	0.9963	0.125
EC102	30	2.480 ± 0.369	0.538 ± 0.038	2.681 ± 0.078	8.942 ± 0.044	0.9956	0.143
MA60	12	7.846 *	0.069 ± 0.004	2.642 ± 0.120	8.015 ± 0.025	0.9928	0.139
MA60	22	—	0.275 ± 0.021	3.969 ± 0.211	9.382 ± 0.279	0.9874	0.191
MA60	30	—	0.528 ± 0.024	3.817 ± 0.111	9.154 ± 0.067	0.9870	0.193
T440	12	17.584 ± 0.689	0.060 ± 0.001	3.879 ± 0.045	8.815 ± 0.094	0.9929	0.120
T440	22	—	0.198 ± 0.004	3.831 ± 0.042	9.123 ± 0.110	0.9922	0.135
T440	30	0.663 ± 0.275 *	0.426 ± 0.010	3.746 ± 0.090	9.445 ± 0.068	0.9969	0.100
T640	12	13.637 ± 4.865	0.055 ± 0.005	3.849 ± 0.037	8.898 (*n* = 1)	0.9902	0.146
T640	22	1.534 *	0.261 ± 0.014	3.685 ± 0.079	9.225 ± 0.056	0.9961	0.106
T640	30	—	0.494 ± 0.008	3.517 ± 0.072	9.182 ± 0.018	0.9870	0.206
SB10	12	8.507 ± 0.702	0.070 ± 0.002	2.827 ± 0.022	9.152 ± 0.124	0.9958	0.113
SB10	22	3.198 ± 0.179	0.335 ± 0.002	2.867 ± 0.054	9.339 ± 0.063	0.9977	0.097
SB10	30	2.295 ± 0.072	0.713 ± 0.007	2.912 ± 0.024	8.888 ± 0.019	0.9937	0.164
T280	12	4.264 ± 0.882 *	0.063 ± 0.000	3.660 ± 0.153	9.444 ± 0.119	0.9969	0.090
T280	22	—	0.281 ± 0.015	3.817 ± 0.142	9.487 ± 0.163	0.9834	0.229
T280	30	0.925 *	0.557 ± 0.029	3.544 ± 0.207	8.912 ± 0.017	0.9916	0.157
M41	12	7.094 ± 0.793 *	0.069 ± 0.004	3.638 ± 0.169	8.996 ± 0.031	0.9903	0.158
M41	22	—	0.306 ± 0.003	3.484 ± 0.022	8.841 ± 0.046	0.9817	0.230
M41	30	1.002 ± 0.035	0.524 ± 0.002	3.698 ± 0.064	8.856 ± 0.033	0.9904	0.163

Values are mean ± standard deviation of three independent curve fits unless otherwise indicated. An asterisk (*) indicates that the lag estimate was obtained from fewer than three fitted curves (*n* = 1 or *n* = 2) and is therefore interpreted descriptively. *R*^2^ is the mean of the individual fits; RMSE was pooled from the residual errors of the three curves.

**Table 2:** Ratkowsky square-root model parameters for the nine strains.

Strain	*b*	95% CI for b	*T*_min_ (°C)	95% CI for *T*_min_	*R* ^2^	RMSE
EC194	0.0275	−0.0156 to 0.0707	1.69	−31.19 to 34.58	0.9850	0.02500
EC4	0.0296	−0.0014 to 0.0606	2.76	−18.14 to 23.65	0.9933	0.01796
EC102	0.0268	−0.0318 to 0.0855	1.66	−44.29 to 47.61	0.9712	0.03399
MA60	0.0257	0.0228 to 0.0287	1.71	−0.69 to 4.11	0.9999	0.00171
T440	0.0226	0.0014 to 0.0438	1.48	−18.41 to 21.38	0.9946	0.01228
T640	0.0261	0.0130 to 0.0393	2.86	−7.15 to 12.87	0.9984	0.00762
SB10	0.0322	0.0259 to 0.0384	3.84	0.16 to 7.53	0.9998	0.00362
T280	0.0276	0.0243 to 0.0308	2.88	0.56 to 5.21	0.9999	0.00187
M41	0.0258	−0.0024 to 0.0541	1.45	−21.74 to 24.65	0.9926	0.01637

**Table 3:** Linearized Arrhenius model parameters for the nine strains.

Strain	ln(*A*)	95% CI for ln(*A*)	*E*_a_ (kJ/mol)	95% CI for *E*_a_	*R* ^2^	RMSE
EC194	33.205	−62.851 to 129.260	84.69	−150.26 to 319.65	0.9545	0.1897
EC4	35.260	−54.229 to 124.748	89.68	−129.21 to 308.57	0.9644	0.1767
EC102	33.616	−80.673 to 147.905	85.83	−193.73 to 365.38	0.9383	0.2257
MA60	31.885	−20.121 to 83.891	81.77	−45.44 to 208.97	0.9852	0.1027
T440	30.355	11.940 to 48.770	78.58	33.54 to 123.63	0.9980	0.0364
T640	34.720	−38.039 to 107.479	89.00	−88.98 to 266.97	0.9758	0.1437
SB10	36.858	−20.637 to 94.352	93.52	−47.12 to 234.15	0.9862	0.1135
T280	34.505	−25.719 to 94.729	88.20	−59.11 to 235.51	0.9830	0.1189
M41	32.189	−47.695 to 112.073	82.44	−112.96 to 277.83	0.9664	0.1578

Confidence intervals are approximate and reflect fits based on three temperatures; RMSE was calculated on the ln(*μ*_max_) scale.

**Table 4:** Predefined strain pairs and focal antimicrobial-susceptibility designations used in the growth-kinetics comparisons.

Predefined Strain Pair	Species	Antimicrobial	Original Phenotype Designation
EC194–EC102	*E. coli*	Ampicillin	EC194 resistant; EC102 susceptible
EC4–EC102	*E. coli*	Tetracycline	EC4 resistant; EC102 susceptible
MA60–T440	*E. faecalis*	Vancomycin	MA60 resistant; T440 susceptible
MA60–T640	*E. faecalis*	Vancomycin	MA60 resistant; T640 susceptible
SB10–T280	*E. faecium*	Vancomycin	SB10 resistant; T280 susceptible
M41–T280	*E. faecium*	Tetracycline	M41 resistant; T280 susceptible

Note: The phenotype designations shown in this table correspond to those used when the strain pairs were originally predefined for the growth-kinetics experiment. The paired isolates are naturally occurring, genetically distinct environmental strains and were not assumed to differ only in antimicrobial-susceptibility phenotype. Archived inhibition-zone measurements, disk contents, current CLSI M100 interpretive criteria, current zone-based classifications, and reference-control information are provided in Supplementary Table S6.

## Data Availability

The data supporting the findings of this study are provided within the article and Supplementary Materials.

## Supplementary Materials

The following supporting information can be downloaded at: https://www.mdpi.com/article/10.3390/antibiotics15090897/s1, Table S1. Pair- and temperature-specific strain × time interaction results from the mixed-design ANOVA; Table S2. Šídák-adjusted comparisons between paired strains at individual sampling times; Table S3. Ratkowsky square-root model outputs; Table S4. Linearized Arrhenius model outputs; Table S5. Identification and isolation source of the bacterial isolates used in the growth-kinetics study; Table S6. Archived disk-diffusion measurements for the focal antimicrobial–strain combinations used in the predefined growth-kinetics comparisons.

## Abbreviations

The following abbreviations are used in this manuscript:

Abbreviation	Definition
AMR	Antimicrobial resistance
CFU	Colony-forming units
MRD	Maximum Recovery Diluent
RMSE	Root mean square error
TSA	Tryptone Soy Agar
TSB	Tryptone Soy Broth
