# Supplementary material for: Pairwise Growth Dynamics of Environmental Escherichia coli and Enterococcus Isolates with Contrasting Antibiotic Susceptibility Phenotypes: Influence of Temperature
Source: Antibiotics (Basel). 2026 Sep 11;15(9):897. doi: 10.3390/antibiotics15090897 (PMC13603210; doi:10.3390/antibiotics15090897)
Supplement: Supplementary file 1 [file antibiotics-15-00897-s001.zip › antibiotics-4540939-supplementary.pdf]

Supplementary Materials

Pairwise Growth Dynamics of Environmental Escherichia coli and Enterococcus Isolates with Contrasting Antibiotic Susceptibility Phenotypes: Influence of Temperature

Table S1. Pair- and temperature-specific strain × time interaction results from the mixed-design ANOVA.

Pair	Focal antimicrobial	T (°C)	F	df1	df2	GG df1	GG df2	GG ε	p (GG-corrected)	Significant
EC194–EC102	AMP	12	12.399	19	76	2.257	9.028	0.119	0.002182	Yes
EC194–EC102	AMP	22	14.604	10	40	1.937	7.750	0.194	0.002441	Yes
EC194–EC102	AMP	30	16.319	15	60	1.345	5.378	0.090	0.006879	Yes
EC4–EC102	TET	12	5.482	19	76	2.110	8.441	0.111	0.028712	Yes
EC4–EC102	TET	22	12.462	10	40	2.609	10.435	0.261	0.00111	Yes
EC4–EC102	TET	30	27.363	15	60	1.398	5.593	0.093	0.00177	Yes
MA60–T440	VAN	12	38.438	9	36	2.385	9.542	0.265	1.89e−5	Yes
MA60–T440	VAN	22	18.462	10	40	1.945	7.778	0.194	0.001154	Yes
MA60–T440	VAN	30	17.364	13	52	1.572	6.288	0.121	0.003444	Yes
MA60–T640	VAN	12	32.784	9	36	2.064	8.256	0.229	0.000112	Yes
MA60–T640	VAN	22	13.554	10	40	2.283	9.131	0.228	0.001528	Yes
MA60–T640	VAN	30	5.490	13	52	1.393	5.573	0.107	0.054768	No
SB10–T280	VAN	12	13.375	9	36	2.277	9.107	0.253	0.001619	Yes
SB10–T280	VAN	22	132.536	10	40	1.946	7.782	0.195	1.02e−6	Yes
SB10–T280	VAN	30	91.602	13	52	3.131	12.524	0.241	7.76e−9	Yes
M41–T280	TET	12	13.878	9	36	2.685	10.739	0.298	0.000623	Yes
M41–T280	TET	22	45.932	10	40	2.533	10.132	0.253	4.83e−6	Yes
M41–T280	TET	30	18.086	13	52	3.001	12.003	0.231	9.50e−5	Yes

Note: The reported F statistics and Greenhouse–Geisser-corrected p-values correspond to the strain × time interaction, which tested whether the temporal pattern of population development differed between the paired strains. df1 and df2 are the uncorrected degrees of freedom; GG df1 and GG df2 are the corresponding Greenhouse–Geisser-corrected degrees of freedom. GG, Greenhouse–Geisser; ε, Greenhouse–Geisser epsilon; AMP, ampicillin; TET, tetracycline; VAN, vancomycin.

Table S2. Šídák-adjusted comparisons between paired strains at individual sampling times

Pair	Focal antimicrobial	T (°C)	Time (h)	Mean first-listed strain	Mean second-listed strain	Difference (first − second)	Unadjusted p	Šídák-adjusted p	Significant
EC194–EC102	AMP	12	0	2.779	2.642	0.137	0.034875	0.50833	No
EC194–EC102	AMP	12	6	2.635	2.800	-0.165	0.011858	0.212249	No
EC194–EC102	AMP	12	12	3.016	3.060	-0.044	0.604286	1	No
EC194–EC102	AMP	12	18	3.269	3.213	0.056	0.256087	0.997306	No
EC194–EC102	AMP	12	24	3.562	3.569	-0.007	0.887282	1	No
EC194–EC102	AMP	12	30	3.989	4.147	-0.158	0.563907	1	No
EC194–EC102	AMP	12	36	4.635	4.700	-0.065	0.059071	0.704105	No
EC194–EC102	AMP	12	42	4.875	5.100	-0.225	0.015384	0.266612	No
EC194–EC102	AMP	12	48	5.329	5.440	-0.111	0.172263	0.977205	No
EC194–EC102	AMP	12	54	5.616	6.037	-0.421	0.001065	0.021091	Yes
EC194–EC102	AMP	12	60	5.848	6.317	-0.469	5.94e−5	0.001188	Yes
EC194–EC102	AMP	12	66	6.447	6.597	-0.150	0.141308	0.952494	No
EC194–EC102	AMP	12	72	7.114	6.833	0.280	0.004058	0.078099	No
EC194–EC102	AMP	12	78	7.525	7.250	0.275	0.001381	0.027267	Yes
EC194–EC102	AMP	12	84	7.757	7.580	0.177	0.078387	0.80458	No
EC194–EC102	AMP	12	90	8.425	7.775	0.650	0.001093	0.021633	Yes
EC194–EC102	AMP	12	96	8.457	7.965	0.492	0.000533	0.010612	Yes
EC194–EC102	AMP	12	102	8.574	8.330	0.244	0.0061	0.115191	No
EC194–EC102	AMP	12	108	8.743	8.525	0.218	0.108297	0.89898	No
EC194–EC102	AMP	12	114	8.730	8.594	0.136	0.225522	0.993972	No
EC194–EC102	AMP	22	0	2.778	2.642	0.136	0.035404	0.327334	No
EC194–EC102	AMP	22	3	3.137	2.647	0.490	0.028626	0.273477	No
EC194–EC102	AMP	22	6	3.580	3.228	0.352	0.011469	0.119169	No
EC194–EC102	AMP	22	9	4.680	4.290	0.390	0.001475	0.016101	Yes
EC194–EC102	AMP	22	12	5.766	5.393	0.373	0.005326	0.057046	No
EC194–EC102	AMP	22	15	7.169	6.509	0.660	0.000161	0.001773	Yes
EC194–EC102	AMP	22	18	7.685	7.663	0.023	0.544276	0.999824	No
EC194–EC102	AMP	22	21	8.280	8.050	0.230	0.008238	0.086978	No
EC194–EC102	AMP	22	24	8.854	8.706	0.148	0.025021	0.243256	No
EC194–EC102	AMP	22	27	8.937	9.000	-0.063	0.379006	0.994704	No
EC194–EC102	AMP	22	30	8.980	9.110	-0.130	0.111146	0.726388	No
EC194–EC102	AMP	30	0	2.778	2.642	0.136	0.035404	0.438272	No
EC194–EC102	AMP	30	1.5	2.781	2.731	0.051	0.200134	0.971928	No
EC194–EC102	AMP	30	3	3.550	3.154	0.396	0.231204	0.985107	No
EC194–EC102	AMP	30	4.5	4.320	3.417	0.904	9.27e−5	0.001482	Yes
EC194–EC102	AMP	30	6	5.490	4.504	0.986	3.22e−5	0.000515	Yes
EC194–EC102	AMP	30	7.5	6.290	5.557	0.733	4.65e−5	0.000744	Yes
EC194–EC102	AMP	30	9	7.050	6.327	0.723	2.07e−5	0.000331	Yes

Pair	Focal antimicrobial	T (°C)	Time (h)	Mean first-listed strain	Mean second-listed strain	Difference (first − second)	Unadjusted p	Šídák-adjusted p	Significant
EC194–EC102	AMP	30	10.5	7.820	6.963	0.857	3.70e−6	5.92e−5	Yes
EC194–EC102	AMP	30	12	8.373	7.637	0.737	0.00068	0.010824	Yes
EC194–EC102	AMP	30	13.5	8.770	8.253	0.517	0.002717	0.04259	Yes
EC194–EC102	AMP	30	15	9.043	8.617	0.427	0.001677	0.026496	Yes
EC194–EC102	AMP	30	16.5	9.070	8.847	0.223	0.015103	0.216119	No
EC194–EC102	AMP	30	18	9.073	8.920	0.153	0.039161	0.472276	No
EC194–EC102	AMP	30	19.5	9.110	8.931	0.179	0.000136	0.002179	Yes
EC194–EC102	AMP	30	21	9.113	8.983	0.130	0.013636	0.197217	No
EC194–EC102	AMP	30	22.5	9.137	8.990	0.147	0.017477	0.245802	No
EC4–EC102	TET	12	0	2.789	2.642	0.146	0.028902	0.443757	No
EC4–EC102	TET	12	6	2.810	2.800	0.010	0.795058	1	No
EC4–EC102	TET	12	12	3.057	3.060	-0.003	0.944772	1	No
EC4–EC102	TET	12	18	3.430	3.213	0.217	0.010612	0.192154	No
EC4–EC102	TET	12	24	3.830	3.569	0.261	0.006264	0.118099	No
EC4–EC102	TET	12	30	4.375	4.147	0.228	0.078924	0.806844	No
EC4–EC102	TET	12	36	4.760	4.700	0.060	0.370617	0.999905	No
EC4–EC102	TET	12	42	5.350	5.100	0.250	0.0132	0.233371	No
EC4–EC102	TET	12	48	5.613	5.440	0.173	0.07612	0.794735	No
EC4–EC102	TET	12	54	5.880	6.037	-0.157	0.022449	0.364978	No
EC4–EC102	TET	12	60	6.320	6.317	0.003	0.961648	1	No
EC4–EC102	TET	12	66	6.760	6.597	0.164	0.024169	0.386953	No
EC4–EC102	TET	12	72	7.283	6.833	0.450	0.001924	0.03779	Yes
EC4–EC102	TET	12	78	7.530	7.250	0.280	0.01613	0.27764	No
EC4–EC102	TET	12	84	7.820	7.580	0.240	0.012312	0.219459	No
EC4–EC102	TET	12	90	8.138	7.775	0.363	0.004161	0.08001	No
EC4–EC102	TET	12	96	8.243	7.965	0.278	0.000838	0.016621	Yes
EC4–EC102	TET	12	102	8.447	8.330	0.117	0.041199	0.568908	No
EC4–EC102	TET	12	108	8.557	8.525	0.032	0.766053	1	No
EC4–EC102	TET	12	114	8.675	8.594	0.082	0.471991	0.999997	No
EC4–EC102	TET	22	0	2.789	2.642	0.146	0.028902	0.275738	No
EC4–EC102	TET	22	3	2.921	2.647	0.274	0.101864	0.693265	No
EC4–EC102	TET	22	6	3.297	3.228	0.069	0.489761	0.99939	No
EC4–EC102	TET	22	9	4.795	4.290	0.505	0.000662	0.007262	Yes
EC4–EC102	TET	22	12	5.651	5.393	0.258	0.021127	0.209342	No
EC4–EC102	TET	22	15	6.652	6.509	0.144	0.071161	0.55604	No
EC4–EC102	TET	22	18	7.587	7.663	-0.076	0.261313	0.964267	No
EC4–EC102	TET	22	21	8.745	8.050	0.695	9.98e−5	0.001097	Yes
EC4–EC102	TET	22	24	9.180	8.706	0.474	0.004156	0.044775	Yes
EC4–EC102	TET	22	27	9.460	9.000	0.460	0.019702	0.196587	No
EC4–EC102	TET	22	30	9.679	9.110	0.569	0.001161	0.012701	Yes
EC4–EC102	TET	30	0	2.789	2.642	0.146	0.028902	0.374522	No

Pair	Focal antimicrobial	T (°C)	Time (h)	Mean first-listed strain	Mean second-listed strain	Difference (first − second)	Unadjusted p	Šídák-adjusted p	Significant
EC4–EC102	TET	30	1.5	2.800	2.731	0.069	0.297225	0.99646	No
EC4–EC102	TET	30	3	3.328	3.154	0.174	0.561554	0.999998	No
EC4–EC102	TET	30	4.5	4.745	3.417	1.329	1.79e−5	0.000286	Yes
EC4–EC102	TET	30	6	5.631	4.504	1.128	8.42e−6	0.000135	Yes
EC4–EC102	TET	30	7.5	6.640	5.557	1.083	8.95e−6	0.000143	Yes
EC4–EC102	TET	30	9	7.634	6.327	1.307	1.55e−6	2.48e−5	Yes
EC4–EC102	TET	30	10.5	8.102	6.963	1.139	1.73e−6	2.77e−5	Yes
EC4–EC102	TET	30	12	8.660	7.637	1.023	0.000166	0.002657	Yes
EC4–EC102	TET	30	13.5	8.985	8.253	0.732	0.001945	0.030676	Yes
EC4–EC102	TET	30	15	9.243	8.617	0.627	0.000749	0.01191	Yes
EC4–EC102	TET	30	16.5	9.330	8.847	0.483	0.001699	0.026844	Yes
EC4–EC102	TET	30	18	9.360	8.920	0.440	0.001532	0.02423	Yes
EC4–EC102	TET	30	19.5	9.490	8.931	0.559	1.19e−6	1.91e−5	Yes
EC4–EC102	TET	30	21	9.513	8.983	0.530	3.74e−6	5.98e−5	Yes
EC4–EC102	TET	30	22.5	9.577	8.990	0.587	2.69e−5	0.00043	Yes
MA60–T440	VAN	12	0	2.752	3.782	-1.030	0.000207	0.00207	Yes
MA60–T440	VAN	12	12	3.130	3.988	-0.857	0.000246	0.00246	Yes
MA60–T440	VAN	12	24	4.222	4.242	-0.020	0.919805	1	No
MA60–T440	VAN	12	36	4.852	4.959	-0.107	0.173942	0.852053	No
MA60–T440	VAN	12	48	5.841	5.643	0.198	0.042644	0.353251	No
MA60–T440	VAN	12	60	6.442	6.687	-0.245	0.003331	0.032818	Yes
MA60–T440	VAN	12	72	7.361	6.943	0.419	0.000599	0.005977	Yes
MA60–T440	VAN	12	84	7.899	7.842	0.056	0.357374	0.987989	No
MA60–T440	VAN	12	96	7.940	8.293	-0.353	0.006407	0.062257	No
MA60–T440	VAN	12	108	7.920	8.680	-0.760	0.000196	0.00196	Yes
MA60–T440	VAN	22	0	3.752	3.782	-0.030	0.722344	0.999999	No
MA60–T440	VAN	22	3	4.713	4.241	0.472	0.001355	0.0148	Yes
MA60–T440	VAN	22	6	5.758	5.052	0.706	0.001683	0.018356	Yes
MA60–T440	VAN	22	9	6.757	5.733	1.023	0.000703	0.007702	Yes
MA60–T440	VAN	22	12	7.490	6.384	1.106	2.93e−6	3.22e−5	Yes
MA60–T440	VAN	22	15	7.867	6.945	0.922	0.000177	0.001943	Yes
MA60–T440	VAN	22	18	8.487	7.422	1.065	1.25e−5	0.000137	Yes
MA60–T440	VAN	22	21	9.006	7.797	1.209	1.18e−6	1.30e−5	Yes
MA60–T440	VAN	22	24	9.243	8.330	0.913	0.005655	0.060477	No
MA60–T440	VAN	22	27	9.410	8.620	0.790	0.003572	0.038598	Yes
MA60–T440	VAN	22	30	9.507	9.137	0.371	0.127645	0.777348	No
MA60–T440	VAN	30	0	3.752	3.782	-0.030	0.722344	1	No
MA60–T440	VAN	30	1.5	4.697	4.162	0.535	0.017683	0.221028	No
MA60–T440	VAN	30	3	5.233	4.808	0.425	0.003278	0.044922	Yes
MA60–T440	VAN	30	4.5	6.296	5.395	0.901	0.02536	0.302061	No
MA60–T440	VAN	30	6	7.060	6.165	0.894	2.39e−5	0.000334	Yes

Pair	Focal antimicrobial	T (°C)	Time (h)	Mean first-listed strain	Mean second-listed strain	Difference (first − second)	Unadjusted p	Šídák-adjusted p	Significant
MA60–T440	VAN	30	7.5	7.853	6.725	1.128	0.000447	0.006246	Yes
MA60–T440	VAN	30	9	8.374	7.548	0.826	0.000298	0.004169	Yes
MA60–T440	VAN	30	10.5	8.693	7.928	0.765	0.000109	0.001531	Yes
MA60–T440	VAN	30	12	8.841	8.567	0.274	0.043003	0.459556	No
MA60–T440	VAN	30	13.5	8.958	8.954	0.004	0.869243	1	No
MA60–T440	VAN	30	15	9.116	9.110	0.007	0.916763	1	No
MA60–T440	VAN	30	16.5	9.217	9.257	-0.040	0.681777	1	No
MA60–T440	VAN	30	18	9.363	9.483	-0.120	0.048262	0.499681	No
MA60–T440	VAN	30	19.5	9.369	9.575	-0.206	0.241928	0.979301	No
MA60–T640	VAN	12	0	2.752	3.765	-1.013	0.000203	0.002027	Yes
MA60–T640	VAN	12	12	3.130	4.035	-0.905	3.61e−5	0.000361	Yes
MA60–T640	VAN	12	24	4.222	4.308	-0.086	0.715006	0.999996	No
MA60–T640	VAN	12	36	4.852	4.906	-0.054	0.503079	0.999082	No
MA60–T640	VAN	12	48	5.841	5.775	0.066	0.361305	0.988704	No
MA60–T640	VAN	12	60	6.442	6.681	-0.238	0.005412	0.052822	No
MA60–T640	VAN	12	72	7.361	6.943	0.419	0.000624	0.006223	Yes
MA60–T640	VAN	12	84	7.899	7.630	0.268	0.012582	0.118926	No
MA60–T640	VAN	12	96	7.940	8.311	-0.371	0.002689	0.026565	Yes
MA60–T640	VAN	12	108	7.920	8.722	-0.802	0.000342	0.00341	Yes
MA60–T640	VAN	22	0	3.752	3.765	-0.013	0.873896	1	No
MA60–T640	VAN	22	3	4.713	4.386	0.328	0.033196	0.310204	No
MA60–T640	VAN	22	6	5.758	5.048	0.710	0.001574	0.017177	Yes
MA60–T640	VAN	22	9	6.757	5.830	0.927	0.00119	0.013007	Yes
MA60–T640	VAN	22	12	7.490	6.476	1.014	6.44e−6	7.08e−5	Yes
MA60–T640	VAN	22	15	7.867	7.395	0.472	0.005271	0.056474	No
MA60–T640	VAN	22	18	8.487	8.330	0.157	0.095038	0.666625	No
MA60–T640	VAN	22	21	9.006	8.783	0.223	0.001047	0.011458	Yes
MA60–T640	VAN	22	24	9.243	9.017	0.227	0.22631	0.940538	No
MA60–T640	VAN	22	27	9.410	9.171	0.239	0.076927	0.58543	No
MA60–T640	VAN	22	30	9.507	9.206	0.301	0.191304	0.903252	No
MA60–T640	VAN	30	0	3.752	3.765	-0.013	0.873896	1	No
MA60–T640	VAN	30	1.5	4.697	3.976	0.721	0.008818	0.11662	No
MA60–T640	VAN	30	3	5.233	4.675	0.559	0.000228	0.003184	Yes
MA60–T640	VAN	30	4.5	6.296	5.850	0.446	0.181166	0.939083	No
MA60–T640	VAN	30	6	7.060	6.645	0.415	0.007207	0.096301	No
MA60–T640	VAN	30	7.5	7.853	7.537	0.317	0.032646	0.371657	No
MA60–T640	VAN	30	9	8.374	7.940	0.434	0.002027	0.028013	Yes
MA60–T640	VAN	30	10.5	8.693	8.260	0.433	0.002653	0.036508	Yes
MA60–T640	VAN	30	12	8.841	8.715	0.126	0.010492	0.137278	No
MA60–T640	VAN	30	13.5	8.958	8.959	-0.001	0.955853	1	No
MA60–T640	VAN	30	15	9.116	9.091	0.025	0.342257	0.997163	No

Pair	Focal antimicrobial	T (°C)	Time (h)	Mean first-listed strain	Mean second-listed strain	Difference (first − second)	Unadjusted p	Šidák-adjusted p	Significant
MA60–T640	VAN	30	16.5	9.217	9.232	-0.015	0.820589	1	No
MA60–T640	VAN	30	18	9.363	9.277	0.087	0.092922	0.744714	No
MA60–T640	VAN	30	19.5	9.369	9.355	0.014	0.93034	1	No
SB10–T280	VAN	12	0	2.826	3.697	-0.872	0.0001	0.001	Yes
SB10–T280	VAN	12	12	3.102	4.089	-0.987	8.65e−5	0.000865	Yes
SB10–T280	VAN	12	24	3.846	5.079	-1.233	2.34e−6	2.34e−5	Yes
SB10–T280	VAN	12	36	4.722	5.769	-1.047	5.65e−5	0.000565	Yes
SB10–T280	VAN	12	48	5.532	6.496	-0.964	1.99e−6	1.99e−5	Yes
SB10–T280	VAN	12	60	6.667	7.217	-0.550	0.010678	0.101793	No
SB10–T280	VAN	12	72	7.200	7.944	-0.744	0.000216	0.002158	Yes
SB10–T280	VAN	12	84	7.974	8.590	-0.616	0.008988	0.086334	No
SB10–T280	VAN	12	96	8.507	9.023	-0.517	0.013884	0.130477	No
SB10–T280	VAN	12	108	9.008	9.343	-0.335	0.002778	0.027432	Yes
SB10–T280	VAN	22	0	2.826	3.697	-0.872	0.0001	0.0011	Yes
SB10–T280	VAN	22	3	2.962	4.662	-1.701	0.000108	0.001184	Yes
SB10–T280	VAN	22	6	3.703	5.489	-1.786	1.89e−6	2.08e−5	Yes
SB10–T280	VAN	22	9	4.977	6.324	-1.347	2.47e−7	2.72e−6	Yes
SB10–T280	VAN	22	12	5.724	7.468	-1.744	9.42e−9	1.04e−7	Yes
SB10–T280	VAN	22	15	6.866	8.227	-1.361	4.22e−5	0.000464	Yes
SB10–T280	VAN	22	18	7.743	8.520	-0.777	1.40e−6	1.54e−5	Yes
SB10–T280	VAN	22	21	8.600	8.760	-0.160	0.015309	0.156079	No
SB10–T280	VAN	22	24	9.088	9.139	-0.051	0.049023	0.42473	No
SB10–T280	VAN	22	27	9.167	9.650	-0.483	0.01891	0.189421	No
SB10–T280	VAN	22	30	9.433	9.766	-0.333	0.012974	0.133812	No
SB10–T280	VAN	30	0	2.826	3.697	-0.872	0.0001	0.0014	Yes
SB10–T280	VAN	30	1.5	3.017	4.152	-1.135	1.22e−5	0.000171	Yes
SB10–T280	VAN	30	3	3.474	4.924	-1.449	1.92e−7	2.69e−6	Yes
SB10–T280	VAN	30	4.5	4.370	5.723	-1.353	0.00022	0.003076	Yes
SB10–T280	VAN	30	6	5.355	6.620	-1.265	2.05e−6	2.86e−5	Yes
SB10–T280	VAN	30	7.5	6.918	7.846	-0.928	9.03e−5	0.001264	Yes
SB10–T280	VAN	30	9	7.783	8.223	-0.440	0.002554	0.035171	Yes
SB10–T280	VAN	30	10.5	8.151	8.433	-0.283	0.076147	0.670058	No
SB10–T280	VAN	30	12	8.530	8.791	-0.260	0.013392	0.172016	No
SB10–T280	VAN	30	13.5	8.809	8.810	-0.001	0.93788	1	No
SB10–T280	VAN	30	15	8.956	8.910	0.046	0.037239	0.412162	No
SB10–T280	VAN	30	16.5	8.975	9.019	-0.044	0.541754	0.999982	No
SB10–T280	VAN	30	18	8.971	8.955	0.016	0.854784	1	No
SB10–T280	VAN	30	19.5	8.977	9.002	-0.026	0.645008	0.999999	No
M41–T280	TET	12	0	3.695	3.697	-0.003	0.970685	1	No
M41–T280	TET	12	12	4.267	4.089	0.178	0.019249	0.176647	No
M41–T280	TET	12	24	4.949	5.079	-0.130	0.006249	0.06076	No

Pair	Focal antimicrobial	T (°C)	Time (h)	Mean first-listed strain	Mean second-listed strain	Difference (first − second)	Unadjusted p	Šídák-adjusted p	Significant
M41–T280	TET	12	36	5.498	5.769	-0.271	0.063647	0.48192	No
M41–T280	TET	12	48	6.347	6.496	-0.149	0.002953	0.02914	Yes
M41–T280	TET	12	60	7.584	7.217	0.368	0.038275	0.323127	No
M41–T280	TET	12	72	8.280	7.944	0.336	0.003587	0.035297	Yes
M41–T280	TET	12	84	8.649	8.590	0.059	0.680326	0.999989	No
M41–T280	TET	12	96	8.865	9.023	-0.158	0.140436	0.779819	No
M41–T280	TET	12	108	8.969	9.343	-0.374	0.002499	0.024708	Yes
M41–T280	TET	22	0	3.695	3.697	-0.003	0.970685	1	No
M41–T280	TET	22	3	4.060	4.662	-0.602	0.002492	0.027068	Yes
M41–T280	TET	22	6	5.104	5.489	-0.386	0.000113	0.001241	Yes
M41–T280	TET	22	9	6.744	6.324	0.420	0.001082	0.01184	Yes
M41–T280	TET	22	12	7.064	7.468	-0.404	8.91e−6	9.80e−5	Yes
M41–T280	TET	22	15	8.021	8.227	-0.205	0.041456	0.37233	No
M41–T280	TET	22	18	8.323	8.520	-0.197	0.020252	0.201527	No
M41–T280	TET	22	21	8.640	8.760	-0.120	0.120145	0.755362	No
M41–T280	TET	22	24	8.832	9.139	-0.307	0.000883	0.009668	Yes
M41–T280	TET	22	27	8.918	9.650	-0.732	0.001165	0.01274	Yes
M41–T280	TET	22	30	8.972	9.766	-0.794	0.000557	0.006105	Yes
M41–T280	TET	30	0	3.695	3.697	-0.003	0.970685	1	No
M41–T280	TET	30	1.5	4.099	4.152	-0.053	0.125134	0.846121	No
M41–T280	TET	30	3	4.771	4.924	-0.153	0.010826	0.141352	No
M41–T280	TET	30	4.5	5.197	5.723	-0.526	0.000952	0.013252	Yes
M41–T280	TET	30	6	6.404	6.620	-0.216	0.032024	0.365977	No
M41–T280	TET	30	7.5	7.170	7.846	-0.677	0.000307	0.004284	Yes
M41–T280	TET	30	9	7.714	8.223	-0.509	0.002246	0.03099	Yes
M41–T280	TET	30	10.5	8.594	8.433	0.160	0.259065	0.984971	No
M41–T280	TET	30	12	8.698	8.791	-0.092	0.160528	0.913685	No
M41–T280	TET	30	13.5	8.619	8.810	-0.192	0.044477	0.471095	No
M41–T280	TET	30	15	8.750	8.910	-0.160	0.063911	0.603323	No
M41–T280	TET	30	16.5	8.773	9.019	-0.245	0.030668	0.353433	No
M41–T280	TET	30	18	8.857	8.955	-0.098	0.329179	0.996263	No
M41–T280	TET	30	19.5	9.169	9.002	0.167	0.005843	0.078761	No

Note: The reported difference is first-listed strain minus second-listed strain. Positive values indicate a higher observed mean population for the first-listed strain at that sampling time. Šídák adjustment was applied over all sampling times within each pair–temperature analysis.

Table S3. Ratkowsky square-root model outputs

A. Parameter estimates and approximate 95% confidence intervals

Strain	b	95% CI b	Tmin (°C)	95% CI Tmin (°C)	R^2	RMSE
EC194	0.0275	-0.0156 to 0.0707	1.69	-31.19 to 34.58	0.9850	0.02500
EC4	0.0296	-0.0014 to 0.0606	2.76	-18.14 to 23.65	0.9933	0.01796
EC102	0.0268	-0.0318 to 0.0855	1.66	-44.29 to 47.60	0.9712	0.03399
MA60	0.0257	0.0228 to 0.0287	1.71	-0.69 to 4.11	0.9999	0.00171
T440	0.0226	0.0014 to 0.0438	1.48	-18.41 to 21.37	0.9946	0.01228
T640	0.0261	0.0130 to 0.0393	2.86	-7.15 to 12.87	0.9984	0.00762
SB10	0.0322	0.0259 to 0.0384	3.84	0.16 to 7.53	0.9998	0.00362
T280	0.0276	0.0243 to 0.0308	2.88	0.55 to 5.21	0.9999	0.00187
M41	0.0258	-0.0024 to 0.0541	1.45	-21.74 to 24.65	0.9926	0.01637

Note: Tmin is the theoretical x-intercept of the fitted relationship $\sqrt{\mu max} = b(T - T_{min})$ and should not be interpreted as an experimentally established minimum growth temperature. Approximate confidence intervals reflect fits based on three temperatures. RMSE is reported on the √μmax scale.

B. Temperature-specific inputs and fit diagnostics

Strain	T (°C)	Mean μmax (log10 CFU/mL/h)	Observed √μmax	Fitted √μmax	Residual
EC194	12	0.071766	0.267891	0.283572	-0.015681
EC194	22	0.352826	0.593992	0.558709	0.035283
EC194	30	0.576409	0.759216	0.778818	-0.019602
EC4	12	0.068957	0.262596	0.273860	-0.011264
EC4	22	0.354662	0.595535	0.570191	0.025344
EC4	30	0.629128	0.793176	0.807256	-0.014080
EC102	12	0.065582	0.256090	0.277414	-0.021323
EC102	22	0.352367	0.593605	0.545628	0.047977
EC102	30	0.538089	0.733546	0.760200	-0.026654
MA60	12	0.069486	0.263603	0.264673	-0.001070
MA60	22	0.274952	0.524359	0.521951	0.002408
MA60	30	0.527709	0.726436	0.727774	-0.001338
T440	12	0.059993	0.244935	0.237231	0.007704
T440	22	0.198450	0.445478	0.462812	-0.017334
T440	30	0.426288	0.652907	0.643277	0.009630
T640	12	0.054673	0.233822	0.238601	-0.004778
T640	22	0.260536	0.510427	0.499676	0.010751
T640	30	0.493595	0.702563	0.708536	-0.005973
SB10	12	0.070123	0.264807	0.262537	0.002271
SB10	22	0.335460	0.579189	0.584297	-0.005109
SB10	30	0.713255	0.844544	0.841706	0.002838

Strain	T (°C)	Mean µmax (log10 CFU/mL/h)	Observed √µmax	Fitted √µmax	Residual
T280	12	0.062641	0.250281	0.251455	-0.001174
T280	22	0.280764	0.529872	0.527230	0.002642
T280	30	0.557085	0.746381	0.747849	-0.001468
M41	12	0.068642	0.261997	0.272266	-0.010269
M41	22	0.306398	0.553532	0.530427	0.023105
M41	30	0.524350	0.724120	0.736956	-0.012836

Table S4. Linearized Arrhenius model outputs

A. Parameter estimates and approximate 95% confidence intervals

Strain	ln(A)	95% CI ln(A)	Apparent Ea (kJ/mol)	95% CI Ea (kJ/mol)	R^2	RMSE
EC194	33.205	-62.851 to 129.260	84.69	-150.26 to 319.65	0.9545	0.1897
EC4	35.260	-54.229 to 124.748	89.68	-129.21 to 308.57	0.9644	0.1767
EC102	33.616	-80.673 to 147.905	85.83	-193.73 to 365.38	0.9383	0.2257
MA60	31.885	-20.121 to 83.891	81.77	-45.44 to 208.97	0.9852	0.1027
T440	30.355	11.940 to 48.770	78.58	33.54 to 123.62	0.9980	0.0364
T640	34.720	-38.039 to 107.479	89.00	-88.97 to 266.97	0.9758	0.1437
SB10	36.858	-20.637 to 94.352	93.52	-47.12 to 234.15	0.9862	0.1135
T280	34.505	-25.719 to 94.729	88.20	-59.11 to 235.51	0.9830	0.1189
M41	32.189	-47.695 to 112.073	82.44	-112.96 to 277.83	0.9664	0.1578

Note: Apparent Ea values were derived from only three temperature points and are therefore treated as descriptive indices of temperature dependence rather than precise thermodynamic quantities. Confidence intervals for the Arrhenius regression parameters were derived from the corresponding linear-regression standard errors. RMSE is reported on the ln(μmax) scale.

B. Temperature-specific inputs and fit diagnostics

Strain	T (°C)	T (K)	1/T (K^{-1})	Mean μmax (log10 CFU/mL/h)	Observed ln(μmax)	Fitted ln(μmax)	Residual
EC194	12	285.15	0.0035069	0.071766	-2.634351	-2.519539	-0.114812
EC194	22	295.15	0.0033881	0.352826	-1.041779	-1.309166	0.267387
EC194	30	303.15	0.0032987	0.576409	-0.550937	-0.398363	-0.152575
EC4	12	285.15	0.0035069	0.068957	-2.674276	-2.567314	-0.106962
EC4	22	295.15	0.0033881	0.354662	-1.036589	-1.285694	0.249105
EC4	30	303.15	0.0032987	0.629128	-0.463420	-0.321277	-0.142143
EC102	12	285.15	0.0035069	0.065582	-2.724450	-2.587844	-0.136606
EC102	22	295.15	0.0033881	0.352367	-1.043082	-1.361224	0.318142
EC102	30	303.15	0.0032987	0.538089	-0.619731	-0.438195	-0.181536
MA60	12	285.15	0.0035069	0.069486	-2.666623	-2.604462	-0.062161
MA60	22	295.15	0.0033881	0.274952	-1.291158	-1.435926	0.144767
MA60	30	303.15	0.0032987	0.527709	-0.639210	-0.556603	-0.082606
T440	12	285.15	0.0035069	0.059993	-2.813525	-2.791514	-0.022011
T440	22	295.15	0.0033881	0.198450	-1.617216	-1.668476	0.051260
T440	30	303.15	0.0032987	0.426288	-0.852641	-0.823391	-0.029250
T640	12	285.15	0.0035069	0.054673	-2.906388	-2.819422	-0.086966
T640	22	295.15	0.0033881	0.260536	-1.345014	-1.547550	0.202536
T640	30	303.15	0.0032987	0.493595	-0.706039	-0.590469	-0.115570
SB10	12	285.15	0.0035069	0.070123	-2.657507	-2.588786	-0.068721
SB10	22	295.15	0.0033881	0.335460	-1.092254	-1.252298	0.160044

Strain	T (°C)	T (K)	1/T (K⁻¹)	Mean μmax (log10 CFU/mL/h)	Observed ln(μmax)	Fitted ln(μmax)	Residual
SB10	30	303.15	0.0032987	0.713255	-0.337916	-0.246593	-0.091323
T280	12	285.15	0.0035069	0.062641	-2.770342	-2.698358	-0.071984
T280	22	295.15	0.0033881	0.280764	-1.270240	-1.437884	0.167643
T280	30	303.15	0.0032987	0.557085	-0.585037	-0.489378	-0.095660
M41	12	285.15	0.0035069	0.068642	-2.678844	-2.583361	-0.095483
M41	22	295.15	0.0033881	0.306398	-1.182871	-1.405242	0.222370
M41	30	303.15	0.0032987	0.524350	-0.645596	-0.518708	-0.126887

Table S5. Identification and isolation source of the bacterial isolates used in the growth-kinetics study.

Strain	Species	Sequence identity (%)	Closest GenBank relative	Isolation source
**EC4**	*Escherichia coli*	100.00	CP121294.1	Mussel flesh, mussel-farming area
**EC102**	*Escherichia coli*	100.00	LR778141.1	Chalastra wastewater-treatment plant, aeration tank
**EC194**	*Escherichia coli*	99.76	OQ933373.1	Chalastra wastewater-treatment plant, influent tank
**SB10**	*Enterococcus faecium*	84.33	CP102534.1	Chalastra wastewater-treatment plant, aeration tank
**M41**	*Enterococcus faecium*	99.50	MT231801.1	Chalastra wastewater-treatment plant, aeration tank
**T280**	*Enterococcus faecium*	99.06	MH348122.1	Mussel-farm water
**MA60**	*Enterococcus faecalis*	99.76	OP156815.1	Mussel flesh, mussel-farming area
**T640**	*Enterococcus faecalis*	98.80	OP156815.1	Sediment
**T440**	*Enterococcus faecalis*	98.80	MW548684.1	Agricultural pumping station

Table S6. Archived disk-diffusion measurements for the focal antimicrobial–strain combinations used in the predefined growth-kinetics comparisons

Strain	Species	Focal antimicrobial	Disk content	Archived inhibition-zone diameter (cm), mean ± SD	Original phenotype designation	Predefined comparison	Current CLSI M100 interpretive criterion (mm)	Current CLSI-based classification	Reference control strain used
**EC194**	*E. coli*	Ampicillin	10 µg	0.00 ± 0.00	Resistant	EC194–EC102	S ≥17; I 14–16; R ≤13	**R**	***E. coli* ATCC 25922**
**EC102**	*E. coli*	Ampicillin	10 µg	1.70 ± 0.00	Susceptible	EC194–EC102	S ≥17; I 14–16; R ≤13	**S**	***E. coli* ATCC 25922**
**EC4**	*E. coli*	Tetracycline	30 µg	2.15 ± 0.25	Resistant	EC4–EC102	S ≥15; I 12–14; R ≤11	**S**	***E. coli* ATCC 25922**
**EC102**	*E. coli*	Tetracycline	30 µg	2.20 ± 0.00	Susceptible	EC4–EC102	S ≥15; I 12–14; R ≤11	**S**	***E. coli* ATCC 25922**
**MA60**	*E. faecalis*	Vancomycin	30 µg	0.00 ± 0.11	Resistant	MA60–T440; MA60–T640	S ≥17; I 15–16; R ≤14	**R**	***E. faecalis* ATCC 29212**
**T440**	*E. faecalis*	Vancomycin	30 µg	1.60 ± 0.00	Susceptible	MA60–T440	S ≥17; I 15–16; R ≤14	**I**	***E. faecalis* ATCC 29212**
**T640**	*E. faecalis*	Vancomycin	30 µg	2.25 ± 0.00	Susceptible	MA60–T640	S ≥17; I 15–16; R ≤14	**S**	***E. faecalis* ATCC 29212**
**SB10**	*E. faecium*	Vancomycin	30 µg	0.00 ± 0.00	Resistant	SB10–T280	S ≥17; I 15–16; R ≤14	**R**	***E. faecalis* ATCC 29212**
**T280**	*E. faecium*	Vancomycin	30 µg	2.50 ± 0.11	Susceptible	SB10–T280	S ≥17; I 15–16; R ≤14	**S**	***E. faecalis* ATCC 29212**
**M41**	*E. faecium*	Tetracycline	30 µg	0.00 ± 0.11	Resistant	M41–T280	S ≥19; I 15–18; R ≤14	**R**	***E. faecalis* ATCC 29212**
**T280**	*E. faecium*	Tetracycline	30 µg	1.28 ± 0.00	Susceptible	M41–T280	S ≥19; I 15–18; R ≤14	**R**	***E. faecalis* ATCC 29212**

Note: Archived inhibition-zone diameters are reported in their original units as mean ± standard deviation (cm), based on six measurements obtained from three diameter measurements on each of two replicate plates. For numerical comparison with the applicable current CLSI M100, 36th edition (2026), disk-diffusion zone-diameter criteria, the archived values were converted to millimetres by multiplying by 10. The "Original phenotype designation" column reports the susceptibility designation used when the strain pairs were predefined for the growth-kinetics experiment. The "Current CLSI-based classification" column reports the category obtained by numerical comparison of the archived zone diameter with the applicable current CLSI M100 criterion and is provided as a current reference interpretation; it does not retrospectively redefine the original experimental strain pairs. *Escherichia coli* ATCC 25922 and *Enterococcus faecalis* ATCC 29212 were used as reference control strains for *E. coli* and *Enterococcus* spp., respectively. R, resistant; I, intermediate; S, susceptible.
